# Construction and validation of a prognostic model for lung adenocarcinoma based on endoplasmic reticulum stress-related genes

**DOI:** 10.1038/s41598-022-23852-z

**Published:** 2022-11-18

**Authors:** Feng Li, Yandie Niu, Wei Zhao, Cheng Yan, Yonghua Qi

**Affiliations:** School of Pharmacy, Key Laboratory of Nano-Carbon Modified Film Technology of Henan Province, Diagnostic Laboratory of Animal Diseases, Xinxiang University, Xinxiang, China

**Keywords:** Cancer, Oncology

## Abstract

Lung adenocarcinoma (LUAD) is one of the most universal types of cancer all over the world and its morbidity continues to rise year by year. Growing evidence has demonstrated that endoplasmic reticulum stress is highly activated in cancer cells and plays a key role in regulating the fate of cancer cells. However, the role and mechanism of endoplasmic reticulum stress in lung adenocarcinoma genesis and development remains unclear. In this research, we developed a prognostic model to predict the overall survival of patients with LUAD utilizing endoplasmic reticulum stress-related genes and screened out potential small molecular compounds, which could assist the clinician in making accurate decisions and better treat LUAD patients. Firstly, we downloaded 419 endoplasmic reticulum stress-related genes (ERSRGs) from Molecular Signatures Database (MSigDB). Secondly, we obtained information about the transcriptome profiling and corresponding clinical data of 59 normal samples and 535 lung adenocarcinoma samples from The Cancer Genome Atlas (TCGA) database. Next, we used the DESeq2 package to identify differentially expressed genes related to endoplasmic reticulum stress. We performed univariate Cox, least absolute shrinkage and selection operator (LASSO), and multivariate Cox regression analysis to establish a prognostic model for LUAD patients based on ERSRGs. Then, we carried out univariate and multivariate independent prognostic analysis of endoplasmic reticulum stress-related gene (ERSRG) score and some clinical traits of lung adenocarcinoma. Additionally, we developed a clinically applicable nomogram for predicting survival for LUAD patients over one, three, and five years. Moreover, we carried out a drug sensitivity analysis to identify novel small molecule compounds for LUAD treatment. Finally, we examined the tumor microenvironment (TME) and immune cell infiltrating analysis to explore the interactions between immune and cancer cells. 142 differentially expressed ERSRGs were identified by using the DESeq2 package. A prognostic model was built based on 7 differentially expressed ERSRGs after performing univariate Cox regression, LASSO regression, and multivariate Cox regression analysis. According to the results of univariate and multivariate independent prognostic analysis, we found ERSRG score can be used as an independent prognostic maker. Using the Kaplan–Meier curves, we found low-risk patients had higher survival probability than high-risk patients in both training set and test set. A nomogram was drawn to predict 1-, 3-, and 5-year survival probability. The calibration curves explained good performance of the model for the prediction of survival. Phenformin, OSU-03012, GSK-650394 and KIN001-135 were identified as the drugs most likely to provide important information to clinicians about the treatment of LUAD patients. A prognostic prediction model was established based on 7 differentially expressed ERSRGs (PDX1, IGFBP1, DDIT4, PPP1R3G, CFTR, DERL3 and NUPR1), which could effectively predict the prognosis of LUAD patients and give a reference for clinical doctors to help LUAD patients to make better treatment tactics. Based on the 4 small molecule compounds (Phenformin, OSU-03012, GSK-650394 and KIN001-135) we discovered, targeting endoplasmic reticulum stress-related genes may also be a therapeutic approach for LUAD patients.

## Introduction

Lung cancer is one of the most common malignant tumors in China and even in the world^1^. According to the statistics of the World Health Organization (WHO), the number of new lung cancer patients worldwide in 2020 alone was 2,206,771, accounting for 11.4% of the new cancer patients, ranking second, deaths were 1,796,144, or 18% of cancer deaths, ranking first^2^. Lung adenocarcinoma, a form of non-small cell lung cancer, is the most common pathological type of lung cancer in humans^3^. Epidemiological studies have reported that adenocarcinoma accounts for approximately 40% of lung cancer cases. Adenocarcinoma is also the most common type of lung cancer among non-smokers, with a high incidence in women and younger patients^4^. Existing treatment methods for lung adenocarcinoma, such as surgical treatment, radiotherapy, and chemotherapy, could hardly meet the survival expectation of patients with advanced lung adenocarcinoma^5^. However, the average 5-year survival rate for patients with lung adenocarcinoma is 15%^1^.Therefore, it is an urgent need to establish new molecular biomarkers and prognostic models to further improve the effectiveness of treatment strategies for patients with LUAD patients.

Endoplasmic reticulum stress is the stress response to the accumulation of a large number of unfolded proteins in the endoplasmic reticulum^6^. Endoplasmic reticulum stress is associated with tumor development and progression via pro-tumorigenic and anti-tumorigenic effects^7^. Recent evidence indicates that endoplasmic reticulum stress has been observed during the development of various tumors, such as breast and lung cancer^8^. Studies show that inhibition of ER stress response led to enhanced survival of the LUAD cells^9^. These findings have confirmed the importance of endoplasmic reticulum stress in LUAD and suggest that differentially expressed endoplasmic reticulum stress-related genes may serve as prognostic markers for LUAD. To our knowledge, there is no prognosis model of differentially expressed endoplasmic reticulum stress-related genes in LUAD that has been established to predict the prognosis of LUAD patients. Therefore, a novel prognostic model with ERSRGs for predicting survival in LUAD is highly needed.

In this study, using the differentially expressed ERSRGs in LUAD, we developed a 7-ERSRGs signature with univariate Cox regression, LASSO regression and multivariate Cox regression analysis in the TCGA database and validated the prognostic model in the GEO database. The immune landscape in LUAD and the potential effects of endoplasmic reticulum stress immunotherapy were explored by tumor microenvironment analysis and immune cell infiltration analysis. Finally, several small-molecule compounds were screened for their potential to be used in LUAD therapy.

## Methods

### Data acquisition

We obtained 419 endoplasmic reticulum stress-related genes from Molecular Signatures Database (MSigDB, http://www.gsea-msigdb.org/gsea/msigdb/, accessed on 15th December 2021). Next, we downloaded the transcriptome profiling data and corresponding clinical data of 59 normal samples and 535 LUAD samples from The Cancer Genome Atlas (TCGA) database (https://portal.gdc.cancer.gov/, accessed on 6th January 2022). Moreover, the microarray and corresponding clinical data of 442 LUAD samples were obtained from the Gene Expression Omnibus GSE72094 dataset with GPL15048 platform (https://www.ncbi.nlm.nih.gov/geo/, accessed on 6th January 2022).

### Identification of differentially expressed endoplasmic reticulum stress-related genes

The DESeq2 package was utilized to calculate differential gene expression of ERSRGs in 59 normal samples and 535 LUAD samples. We set false discovery rate (FDR) < 0.05 and|log2 (FC), fold change in log2|> 0.585 as cutoff criteria to obtain differentially expressed ERSRGs. The volcano plots of 142 differentially expressed ERSRGs were constructed using the OmicStudio tools (http://www.omicstudio.cn/tool). Networks of protein–protein interactions (PPIs) were created by using the STRING database (http://string-db.org). Top 10 hub genes identified by analyzing protein–protein interaction networks using Cytohubba, a plug-in for Cytoscape. Venn diagrams of top 10 hub genes in two algorithms (MCC and EPC) were constructed using TBtools software.

### GO and KEGG functional enrichment analyses

Gene Ontology (GO) and Kyoto Encyclopedia of Genes and Genomes (KEGG) biological process enrichment of differentially expressed ERSRGs was carried out by R statistical software containing packages of “clusterProfiler”, “org.Hs.eg.db”, “enrichplot”, “ggplot2” and “GOplot”. GO results covered cellular component (CC), biological process (BP), and molecular function (MF).

### Identification of prognostic gene signatures

A training set of 535 LUAD samples was taken from the TCGA cohort. We used the survival package (http://bioconductor.org/packages/survival/) in R4.1.2 to perform a univariate Cox regression analysis on ERSRGs from a training set to examine the association between gene expression levels and patients’ survival. A hazard ratio (HR) and p-value were calculated for each ERSRG. A further analysis of the ERSRGs with p < 0.05 was undertaken. The number of ERSRGs was further reduced and the degree of collinearity between ERSRGs was eliminated using LASSO Cox regression analysis. Eventually, based on the results of univariate Cox regression analysis, we analyzed the data using multivariate Cox regression analysis.

### Construction and validation of a prognostic model

The ERSRG score of all samples in the train set were calculated according to the following equation based on the results of multivariate Cox regression analysis.

$$ {\text{Risk}}\,{\text{score}} = {\text{Coef}}_{1} \times {\text{X}}_{1} + {\text{Coef}}_{2} \times {\text{X}}_{2} + \cdots + {\text{Coef}}_{n} \times {\text{X}}_{n} $$

Coef_n_ represents the risk coefficient of ERSRGs obtained from multivariate Cox regression analysis, X_n_ represents the expression of corresponding ERSRGs. Simultaneously, we divided all the LUAD patients in the train set into high-risk (ERSRG score > median value) group and low-risk group (ERSRG score < median value) based on the median risk score. Kaplan–Meier survival plots were obtained using the R package “survival”. ROC curves were generated using the “timeROC” package to test the model’s prognostic accuracy. The test set was made up of samples from the GEO database. Using the same formula used for the train set, we calculated risk scores for patients in the GEO cohort. To investigate whether ERSRG score in LUAD patients could be an independent prognostic factor, we performed univariate and multivariate Cox regression analyses in the train set and test set. Age, gender, stage, Covariates included age, gender, stage, T, M, N, and ERSRG score. We considered p < 0.05 to be statistically significant.

### The construction of nomogram and calibration curves

We created a nomogram and calibration plots in R using the "rms" package. The nomograms were calculated to predict 1, 3, and 5-year survival rates for LUAD patients. The calibration curves visualized the differences between actual 1, 3, and 5-year survival rates and predicted overall survival.

### Drug sensitive analysis

Based on Genomics of Drug Sensitivity in Cancer (GDSC, https://www.cancerrxgene.org/), the difference in drug sensitivity between high-risk and low-risk groups in LUAD was predicted using the “pRRophetic” package in R version (4.1.2). In order to make comparisons, we used half of the maximum inhibitory concentration (IC50). Small molecule compounds with p value < 0.05 were selected. We then visualized two-dimensional drug conformations on the PubChem website (https://pubchem.ncbi.nlm.nih.gov/).

### Tumor microenvironment analysis

The ImmuneScore and StromalScore of each LUAD patient were calculated using “estimate package”. A higher ImmuneScore or StromalScore indicates that more immune cells or stroma were expelled within tumor microenvironment (TME)^10^. Kaplan–Meier curves were plotted using the R survival package to predict the survival difference between low- and high-risk groups of LUAD patients.

### Immune cell infiltration analysis between high-risk group and low-risk group

We performed CIBERSORT to study the tumor immune microenvironment of LUAD patients. CIBERSORT is a method for identifying 22 immune-related cell subsets, including naive and memory B cells, 7 types of T cells, myeloid cells, NK cells, and plasma cells^11^. The difference in immune cells between high-risk and low-risk groups was visualized using a bar plot. For the following analysis of differential immune infiltration levels between high-risk and low-risk groups, only samples with a CIBERSORT p of 0.05 were considered^12^.

### Statistical analyses

R version 4.1.2 was used to perform all statistical analyses. The significance level for statistical analysis was set to p < 0.05.

## Results

### Identification of differential gene expression of ERSRGs

**Figure 1:**
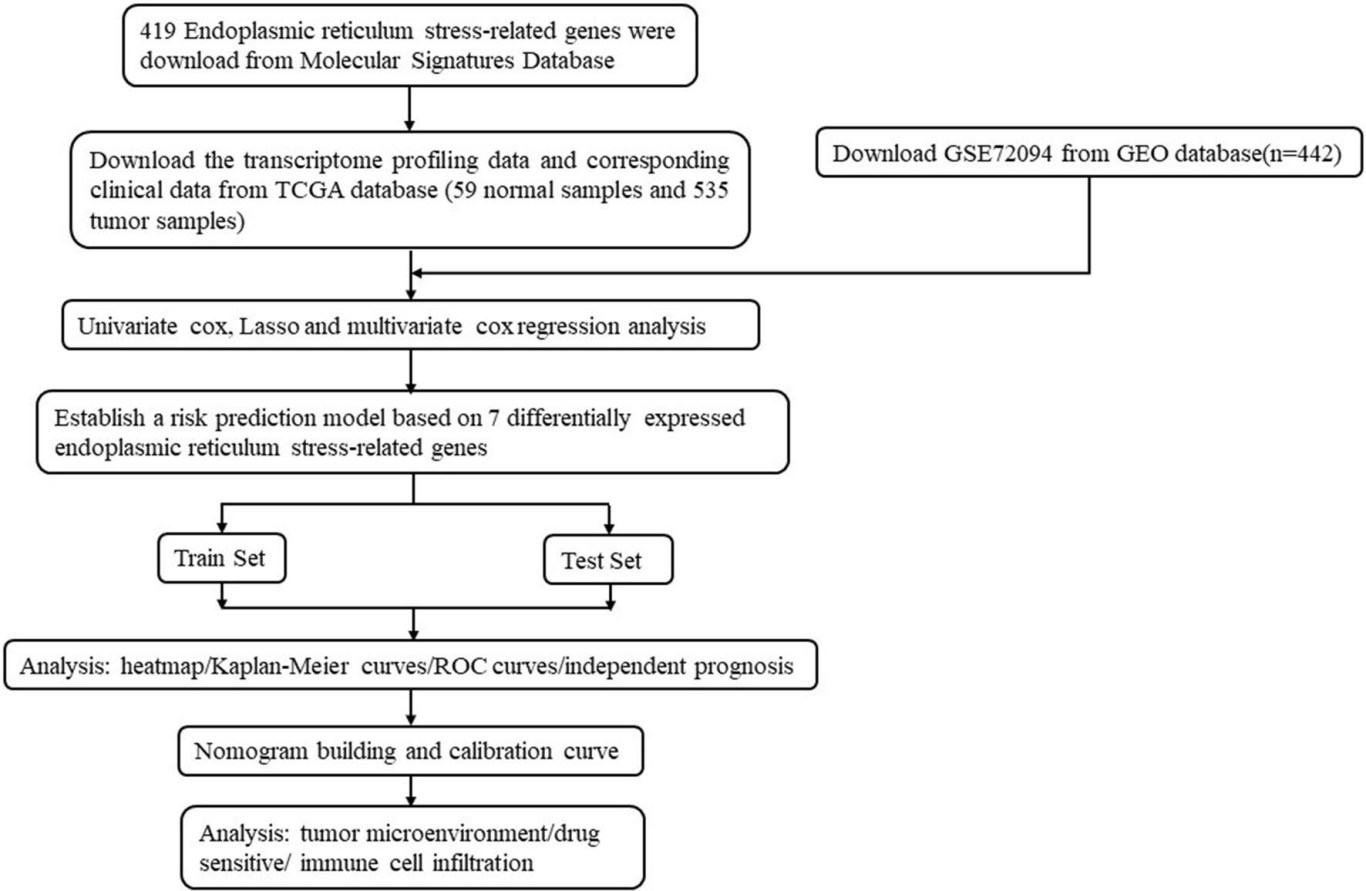
The workflow of our study.

**Figure 2:**
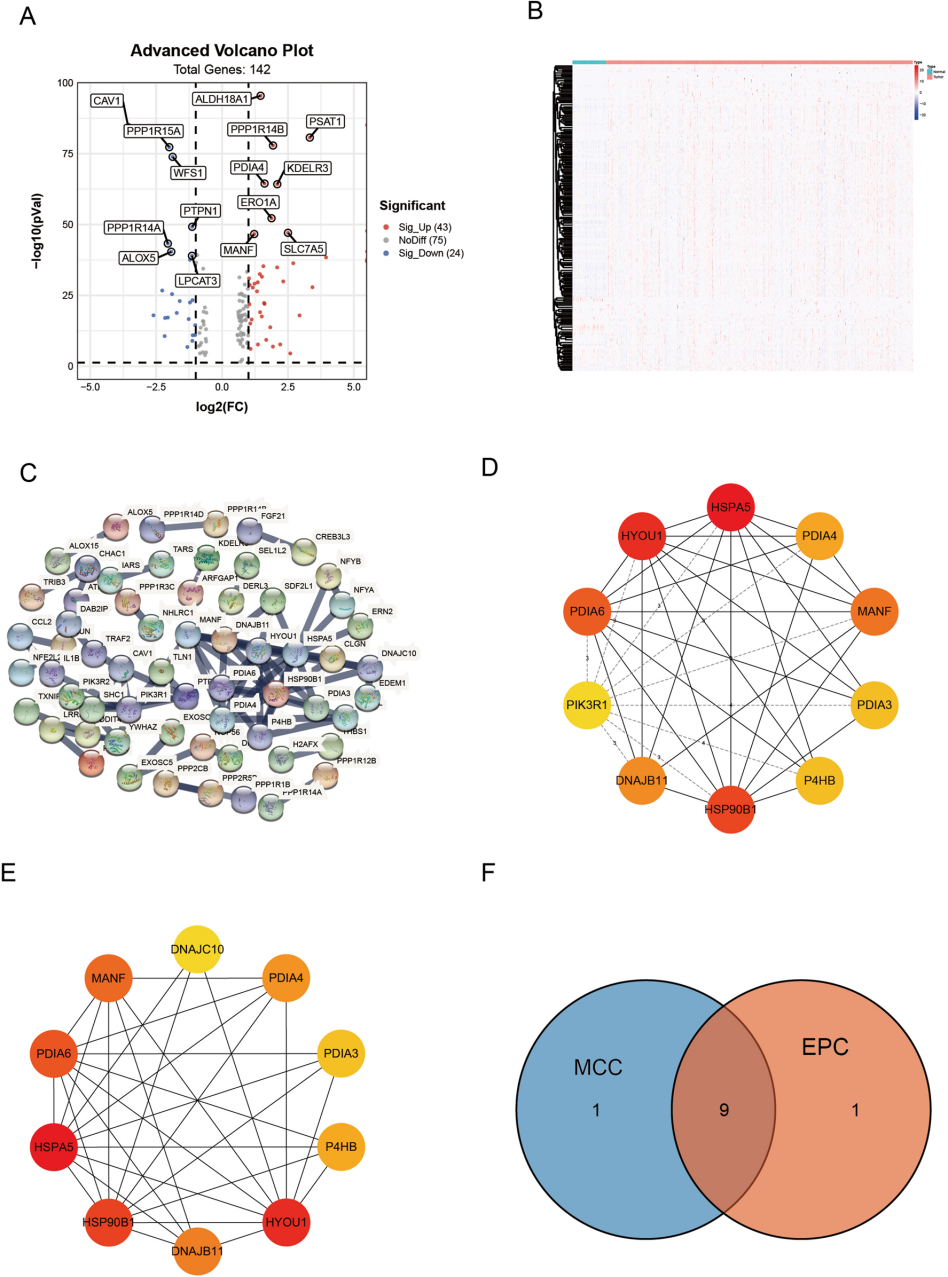
Differentially expressed endoplasmic reticulum stress-related genes (ERSRGs). (**A**) Volcano plot revealed 142 differentially expressed ERSRGs between normal samples and LUAD samples. (**B**) The heatmap displays the expression levels of 142 differentially expressed ERSRGs in normal and LUAD samples. (**C**) Protein–protein interaction (PPI) network of 142 differentially expressed ERSRGs. (**D**) Top 10 hub genes were identified by maximal clique centrality (MCC) method. (**E**) Top 10 hub genes were identified by edge percolated component (EPC) method. (**F**) The Venn diagram showed that 9 of top 10 hub genes screened by MCC method and EPC method were the same.

The overall workflow of this study was shown in Fig. 1. We first obtained the expression profiles containing 59 normal samples and 535 LUAD samples from TCGA database and gathered 419 endoplasmic reticulum stress-related genes from Molecular Signatures Database (MSigDB). 142 differentially expressed ERSRGs were screened out between the normal samples and tumor samples with FDR < 0.05 and |log2 (FC), fold change in log2|> 0.585 (Supplementary Table 1). The volcano plot demonstrated that 24 ERSRGs were significantly downregulated, while 43 ERSRGs were upregulated in LUAD patients (Fig. 2A). Most differentially expressed ERSRGs were enriched in tumor samples according to the heatmap (Fig. 2B). These differentially expressed ERSRGs interacted with each other forming an expressed endoplasmic reticulum stress network as measured by STRING (Fig. 2C). Top 10 hub genes were identified by maximal clique centrality (MCC) method (Fig. 2D) and edge percolated component (EPC) method (Fig. 2E) based on the results of PPIs. The Venn diagram showed that 9 of top 10 hub genes screened by MCC method and EPC method were the same (Fig. 2F).

### Enrichment analysis of ERSRGs

141 differentially expressed ERSRGs were highly enriched in endoplasmic reticulum stress, topologically incorrect protein, unfolded protein, ERAD pathway and intrinsic apoptotic signaling pathway in response to endoplasmic reticulum stress in the aspect of biological process (BP). In the context of cellular components (CC), these genes were more prevalent in endoplasmic reticulum lumen, endoplasmic reticulum protein—containing complex, endoplasmic reticulum chaperone complex, protein serine/threonine phosphatase complex and phosphatase complex. In terms of molecular functions (MF), these genes were more prevalent in protein phosphatase binding, phosphatase binding, phosphatase regulator activity, protein phosphatase regulator activity, protein disulfide isomerase activity (Supplementary Fig. 4A). Moreover, analysis of KEGG revealed that the differentially expressed ERSRGs were mainly involved in signaling pathways such as endoplasmic reticulum stress, ERAD pathway, intrinsic apoptotic signaling pathway in response to endoplasmic reticulum stress (Supplementary Fig. 4B). In summary, these data suggest that these ERSRGs are involved in other biological processes as well as endoplasmic reticulum stress.

### Construction of a prognostic ERSRG signature of LUAD in the train set

**Figure 3:**
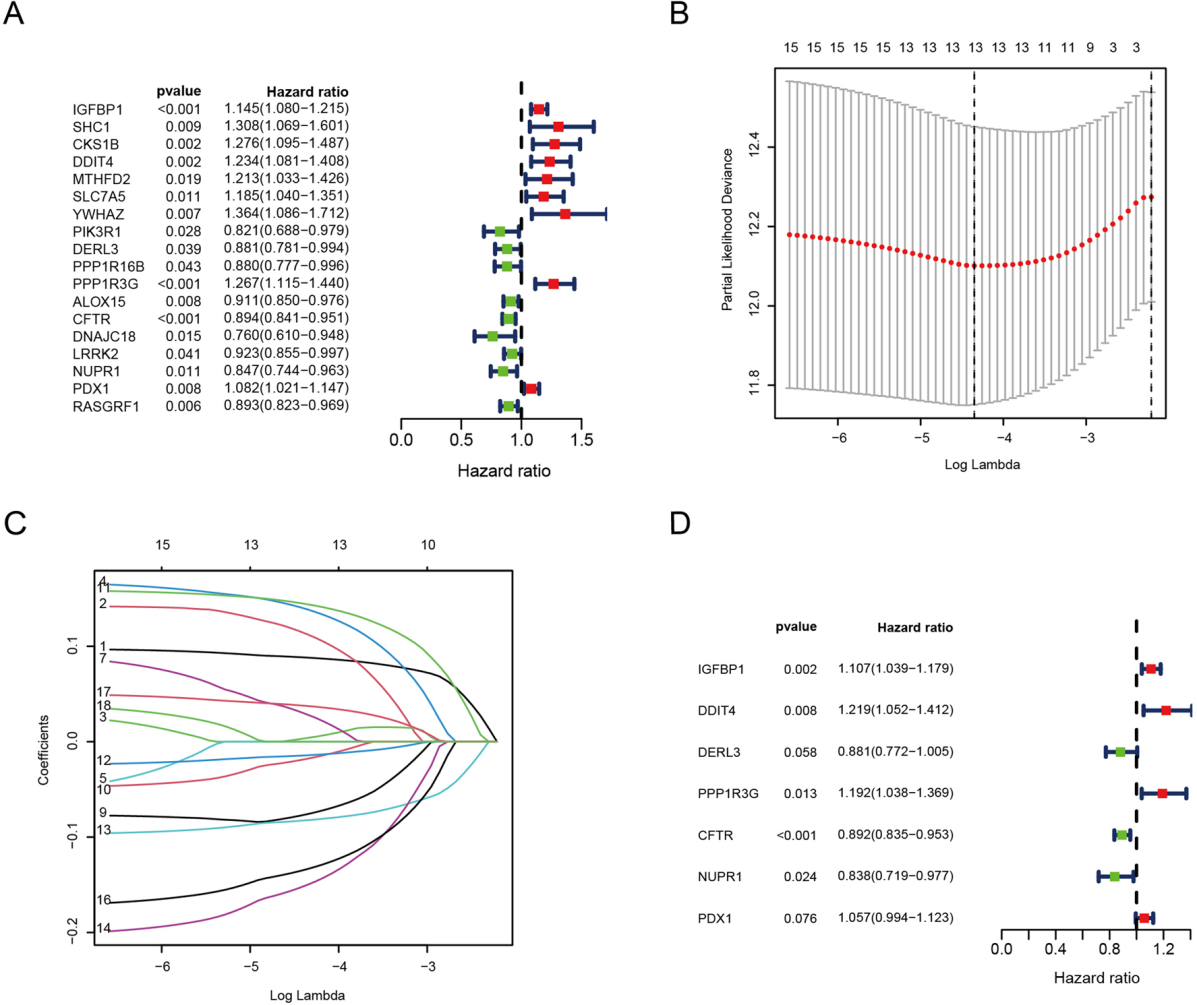
Identification of ERSRGs in LUAD. (**A**) Univariate cox analysis identified 18 prognostic genes. (**B**) Optimal parameter (lambda) screening in the LASSO model. (**C**) The coefficients of each lambda were determined. (**D**) 7 ERSRGs were identified using multivariate Cox regression analysis.

In order to construct the ERSRG prognostic model, we first analyzed all ERSRGs in LUAD using univariate Cox regression analysis. During the screening process, 18 ERSRGs were identified, comprising nine potentially risky genes and nine potentially protective genes (Fig. 3A). On the basis of the univariate Cox regression, we then performed LASSO regression analysis (Fig. 3B,C). Finally, we performed multivariate Cox regression analysis and screened out 7 ER stress-related genes including 3 potential risk genes and 4 potential protective gene (Fig. 3D).

**Figure 4:**
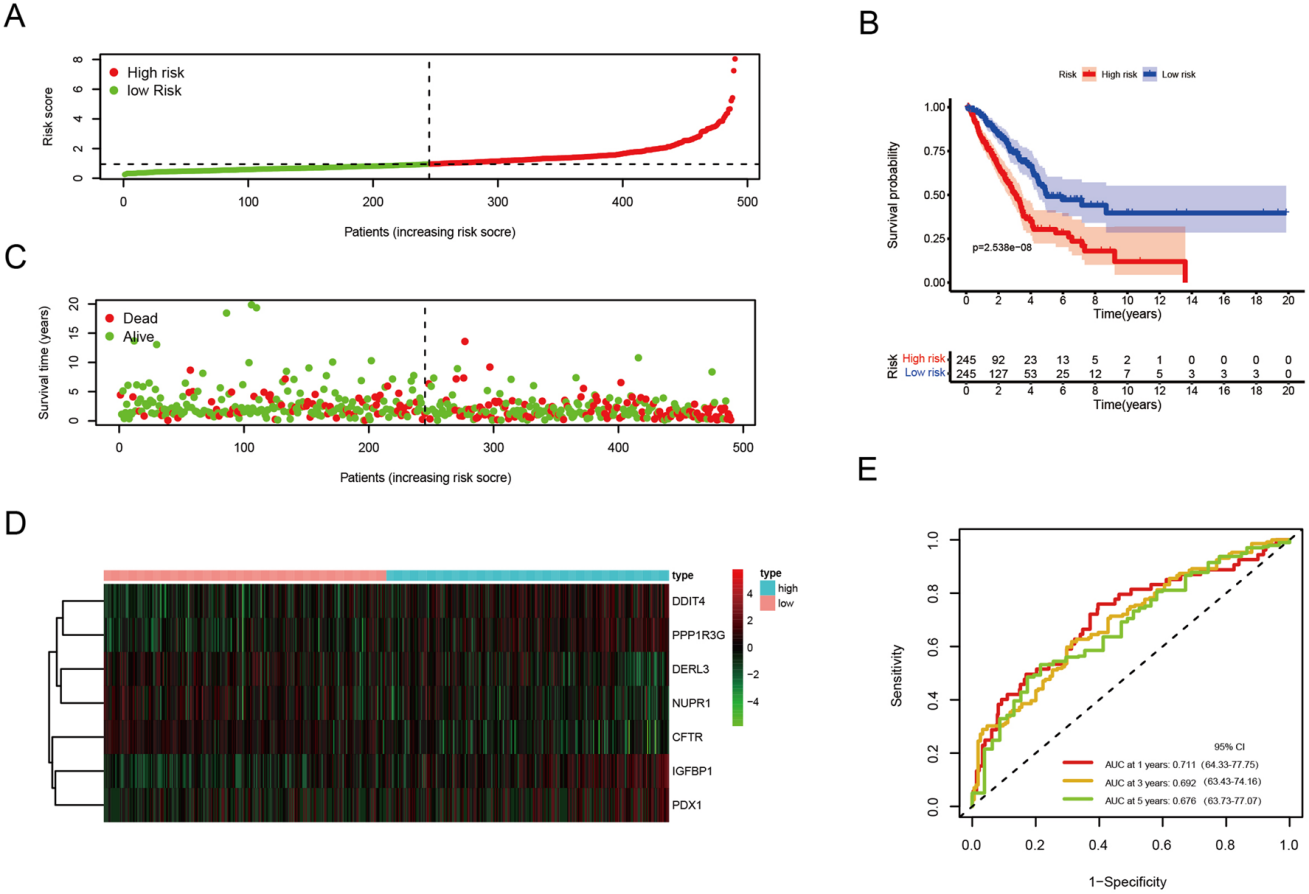
Construction of a prognostic model based on 7 ERSRGs in the training set. (**A**) Risk score plot. (**B**) Curves of Kaplan–Meier survival for high-risk and low-risk patients. (**C**) The survival status of each LUAD patients. (**D**) Heatmap of 7 model ERSRGs. (**E**) ROC curves for prognostic model.

Next, we tested if the expression of these 7 ERSRGs was correlated with the prognosis of LUAD patients. We found that the high expression of DDIT4 (p < 0.001), PDX1 (p = 0.044), PPP1R3G (p < 0.001) had a worse prognosis (Supplementary Figs. 5, 6). Next, we will examine whether the ERSRG signature can be used to predict overall survival (OS) of LUAD. For each LUAD patient, the following formula was used to calculate the ERSRG score: risk score = 0.101362 × IGFBP1 + 0.197865 × DDIT4 + (− 0.127129) × DERL3 + 0.175840 × PPP1R3G + (− 0.114585) × CFTR + (− 0.176282) × NUPR1 + 0.055218 × PDX1. LUAD patients were first categorized into “high risk” (n = 245) and “low risk” (n = 245) groups according to the median value of ERSRG score (Fig. 4A). A higher score was associated with a worse prognosis in LUAD patients (Fig. 4C). The heatmap in Fig. 4D shows the differences between groups in expression levels of the 7 ERSRGs. Additionally, the survival curves indicated that patients in the low-risk group had a significantly higher probability of survival than the high-risk group (p < 0.05) (Fig. 4B). The area under the curve (AUC) for 1-, 3-, and 5-year OS were 0.711, 0.692, and 0.676, respectively (Fig. 4E). These findings suggested that ERSRG signatures in our model may be of benefit to the prognosis prediction of patients with LUAD.

### Validation of the risk score of ER stress-related gene signature in a GEO test set

**Figure 5:**
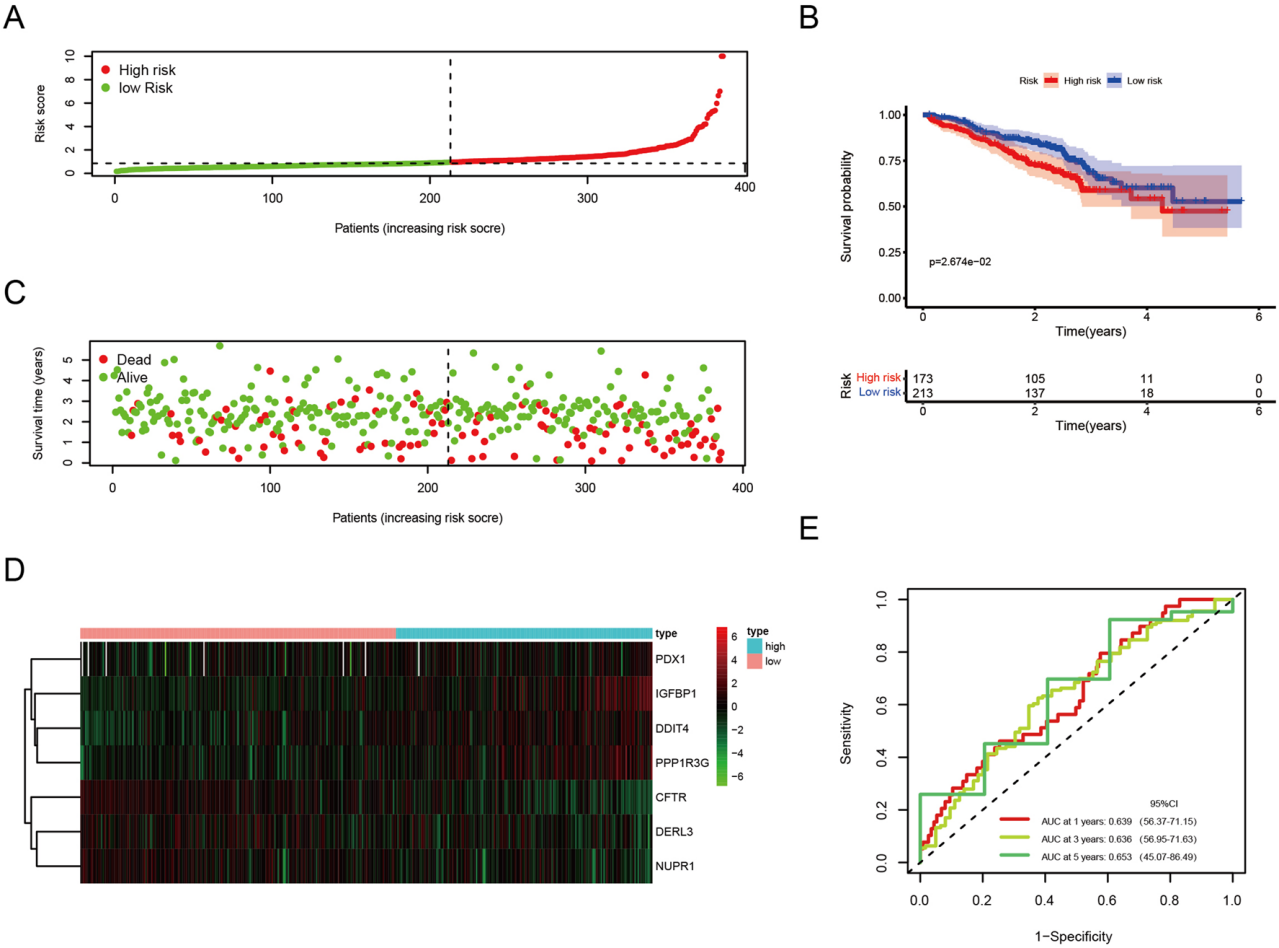
Validation of a prognostic model based on 7 ERSRGs in the test set. (**A**) Risk score plot. (**B**) Curves of Kaplan–Meier survival for high-risk and low-risk patients. (**C**) The survival status of each LUAD patients. (**D**) Heatmap of 7 model ERSRGs. (**E**) ROC curves for the prognostic model.

The prognostic and predictive role of an ERSRG signature was further validated using two more GEO cohorts as a test set, which were calculated using the same formula as the train set. The LUAD patients in the test set were classified as high-risk group (n = 157) and low-risk group (n = 229) based on the median value (Fig. 5A), and a higher ERSRG score predicted a poorer outcome for the patients (Fig. 5C). Visualizing the different expression levels of the 7 ERSRGs in the test groups was achieved with a heatmap (Fig. 5D). As in the train set, high-risk groups in the test set had a worse prognosis than low-risk groups (p < 0.05) (Fig. 5B). The area under the curve (AUC) for 1-, 3-, and 5-year OS were 0.639, 0.636, and 0.653, respectively (Fig. 5E).

### Independent prognostic indicator of the prognostic risk model

**Figure 6:**
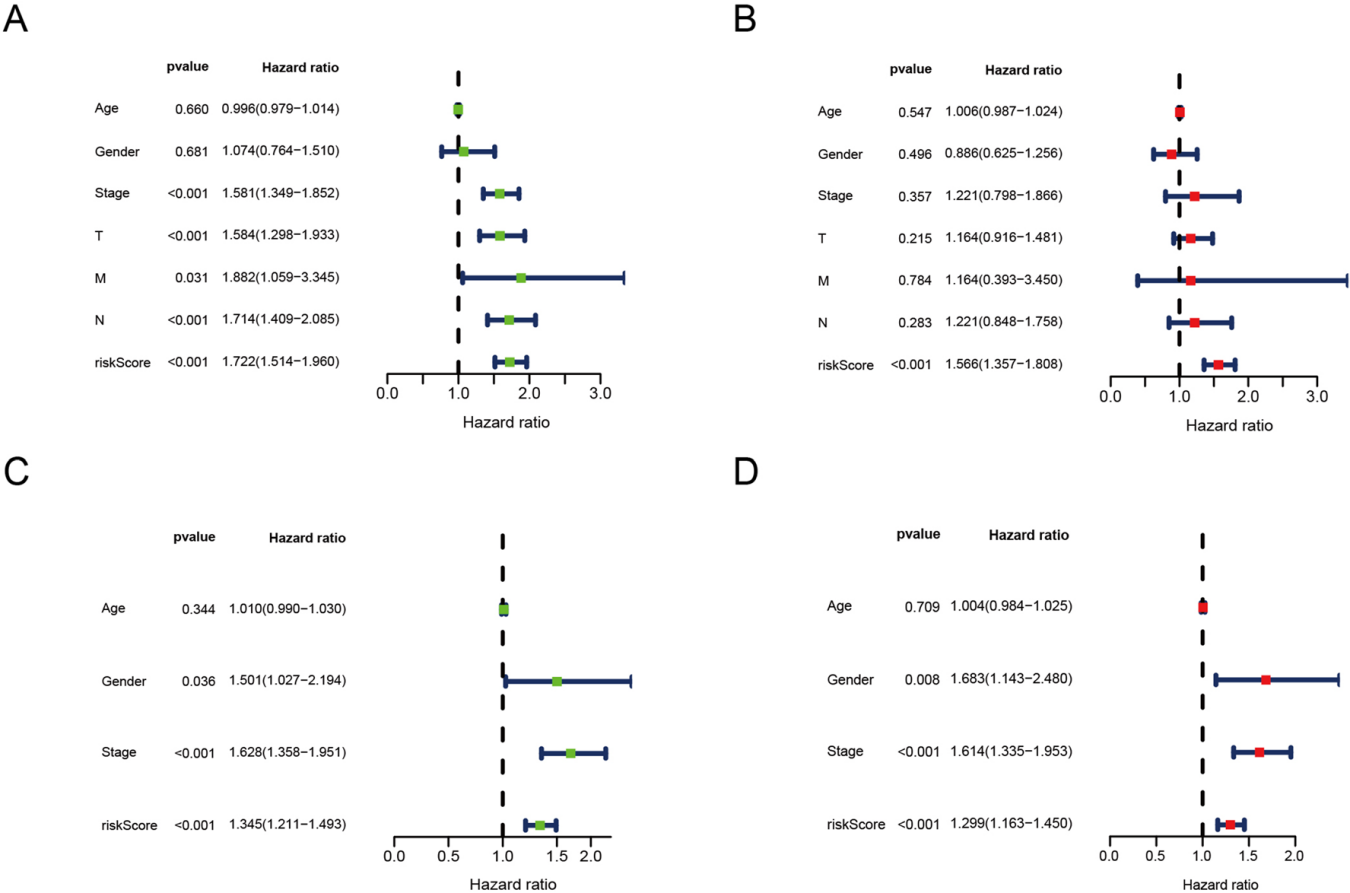
Independent prognosis analysis in the training set and test set. Univariate (**A**) and multivariate (**B**) independent prognosis analysis in the train set. Univariate (**C**) and multivariate (**D**) independent prognosis analysis in the test set.

We conducted independent prognosis analyses in the train set and test set to confirm that ERSRG score can be used as an independent predictor for LUAD patients’ survival. Univariate Cox and multivariate Cox regression analysis of the train set shows that the ERSRG score was meaningful in predicting OS of LUAD patients (Fig. 6A,B). ERSRG score also served as an independent predictor of LUAD patients’ survival in the test set (Fig. 6C,D). Furthermore, we also explored the relationship between clinical characteristics and risk scores. Results indicated that the risk score level was significantly higher in the male subgroup and M1 subtype compare with the female group and M0 subtype, respectively (Supplementary Figs. 7A, B). In the pathologic N-stage subgroup, risk score levels were increased considerably in the N1 and N2 subtypes relative to the N0 subtype (Supplementary Fig. 7C). In the pathologic stage subgroup, risk score levels were statistically significant between Stage1 and other subtypes (Stage2 subtype, Stage3 subtype, Stage4 subtype) (Supplementary Fig. 7D). In the pathologic T-stage subgroup, risk score levels were increased considerably in the T1, T2 and T3 subtypes relative to the T0 subtype (Supplementary Fig. 7E). The results of these studies indicated that our model was capable of predicting prognosis and serving as a biomarker in addition to conventional clinical classification.

### Construction of nomogram and calibration curves

**Figure 7:**
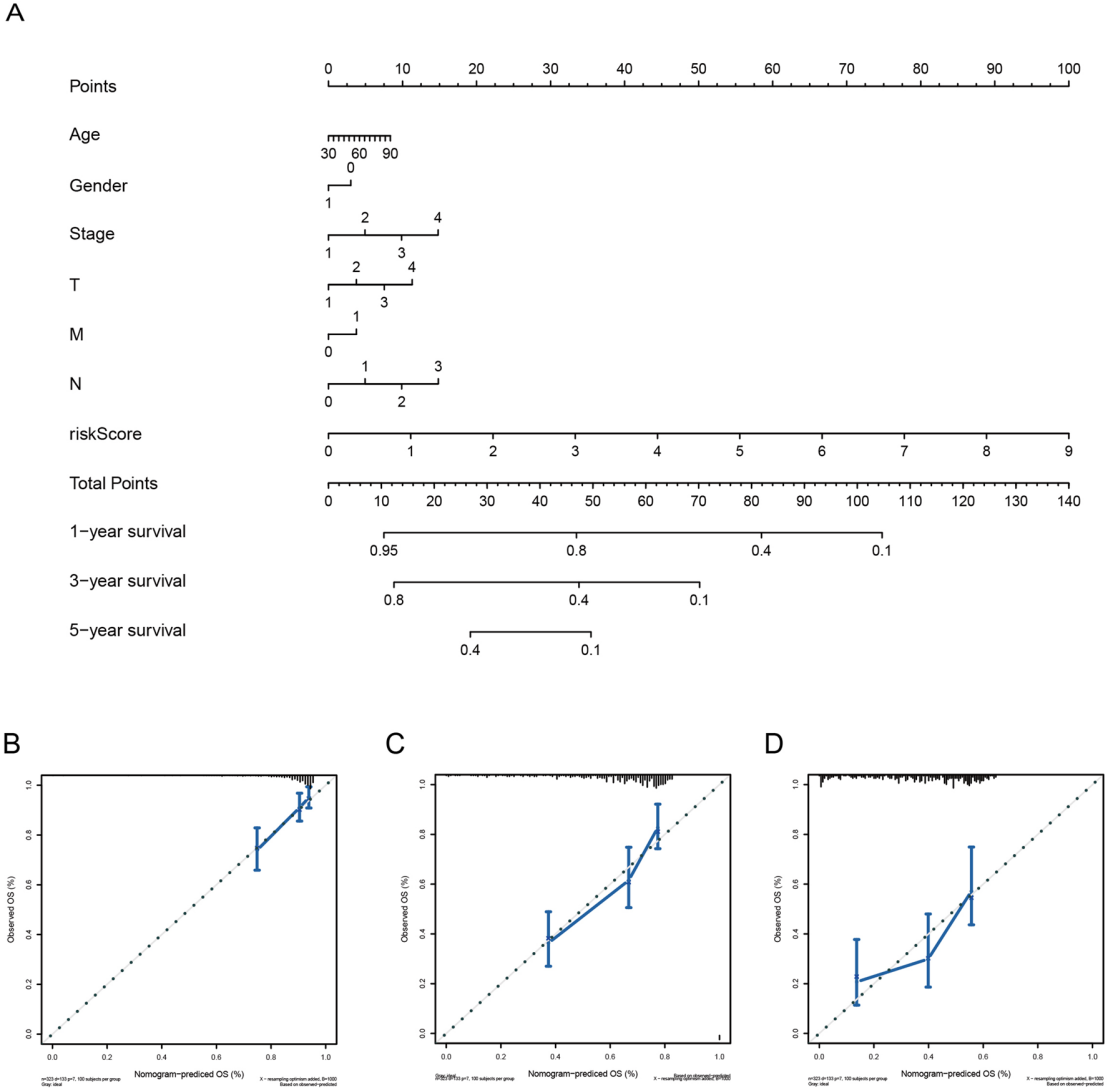
Nomogram and calibration curves. Nomogram (**A**) and calibration curves (**B**–**D**) of LUAD patients for predicting OS at 1 year, 3 years, and 5 years.

To provide clinicians with a more quantitative way of predicting the prognosis of LUAD patients, we developed a nomogram with a number of variables, including age, gender, stage, T, M, N and risk score (Fig. 7A). The nomogram was used to estimate survival probabilities for 1, 3, and 5 years. Moreover, we constructed calibration curves, which confirmed that the predicted and actual survival rates matched with 1, 3, and 5 years (Figs. 7B–D). These findings indicate that the nomogram with the ERSRG scores can accurately predict the OS of LUAD patients.

### Results of drug sensitivity

**Figure 8:**
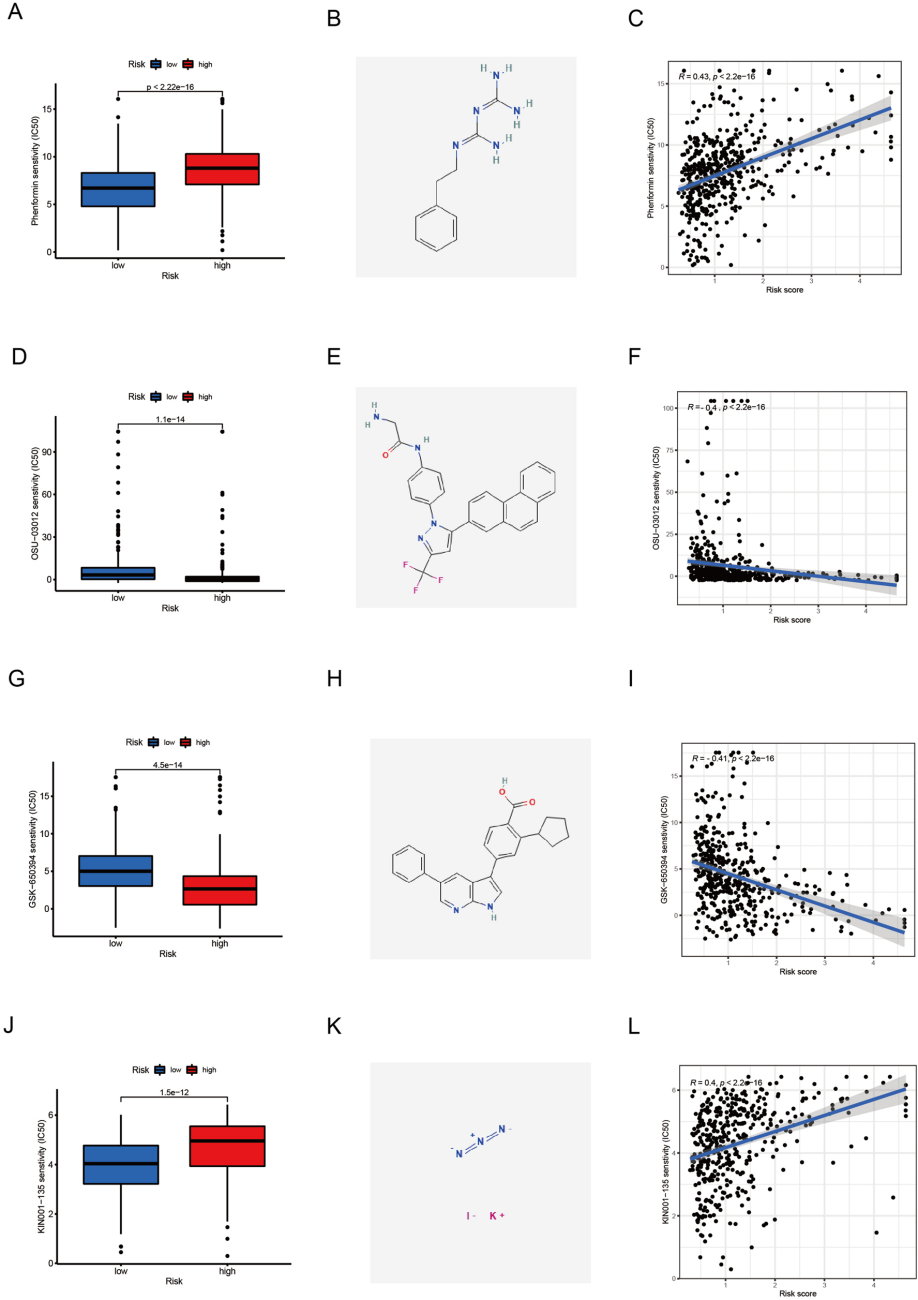
Drug sensitive analysis. Boxplots of (**A**) Phenformin, (**D**) OSU-03012, (**G**) GSK-650394, (**J**) KIN001-135. (**B**, **E**, **H**, **K**) The 2D conformations of the 4 compounds. (**C**, **F**, **I**, **L**) The correlation of the 4 compounds between risk score and drug sensitivity.

To further explore the potential applications of our prognostic model in LUAD therapy, we evaluated differences in chemotherapy sensitivity between the low-risk and high-risk groups by analyzing their IC50s. Patients in the high-risk group responded better to OSU-03012 (Fig. 8D), GSK-650394 (Fig. 8G), whereas patients in the low-risk group responded better to Phenformin (Fig. 8A), KIN001-135 (Fig. 8J). We also visualized the 2D conformation of these four compounds with the greatest differences in sensitivity between the high-risk and low-risk groups (Fig. 8B,E,H,K). The study showed that the model may be used as a predictor of drug sensitivity for the treatment of LUAD. The sensitivity of Phenformin and KIN001-135 were positively correlated with their risk scores (Fig. 8C,L). The sensitivity of OSU-03012 and GSK-650394 were negatively correlated with their risk scores (Fig. 8F,I). These findings revealed that the 4 small molecular compounds we screened may be good anticancer drugs to treat LUAD patients.

### Tumor microenvironment and immune cell infiltration analysis

In our study, we evaluated the relationship between our prognostic model and immune status of patients with LUAD. The ImmuneScore and StromalScore were significantly higher in the low-risk group than in the high-risk group (Supplementary Fig. 1A, B). The Kaplan–Meier curves revealed that LUAD patients who had a high stromal and immune score had a lower chance of survival (Supplementary Fig. 1C, D). The barplot and heatmap were used to illustrate the percentage of each type of immune cell between the high-risk and low-risk groups (Supplementary Fig. 2A, B). Analysis of immune cell infiltration showed a significant difference between the high- and low-risk groups in terms of the types of infiltrating cells (Supplementary Fig. 2C). LUAD patients in the high-risk group had a higher level of NK cells resting, Macrophages M0, and Macrophages M1 compared with the low-risk group. Based on the tight relation between immunological features and LUAD prognosis, we investigated the impact of the risk score model on immune cell infiltration. Macrophages M0, Macrophages M1, NK cells resting, and T cells CD4 memory activated were positively related to the risk score, while B cells naïve, Dendritic cells resting, Mast cells resting, Monocytes, T cells CD4 memory resting were negatively related to risk score (Supplementary Fig. 3A-I). These findings suggested that our prognostic model was closely related to immunity, which could guide treating LUAD patients.

### Correlation of immune cells proportion with seven-ERSRG expression

In this study, we further confirmed the correlation between seven-ERSRG expression and immune cells in LUAD samples. The results showed that lots kinds of immune cells were correlated with the expression of seven-ERSRG (Supplementary Figs. 8–14). Among them, the expression of each ERSRG was correlated with Monocytes, which indicated that Monocytes might play a crucial role in LUAD. According to the above results, we can conclude that the levels of seven-ERSRG expression affected immune activity.

## Discussion

Lung adenocarcinoma (LUAD) is associated with significant morbidity and mortality^13^. There has been an increase in the incidence of lung adenocarcinoma in recent years, and despite using a variety of treatment methods, the mortality rate remains high^14^. Moreover, LUAD has a poor prognosis and inadequate screening methods, which result in a low clinical cure rate^15^. Therefore, a novel biomarker must be developed to predict the prognosis of LUAD and provide treatment targets.

The endoplasmic reticulum capacity to handle protein biogenesis is overtaxed, resulting in an accumulation of improperly folded proteins in this compartment and a situation called endoplasmic reticulum stress^16^. Stress in the endoplasmic reticulum triggers an adaptive response called the unfolded protein response (UPR). As long as ER stress persists, UPR triggers cell death^17,18^. Research showed that abnormal activation of the endoplasmic reticulum stress sensor and its downstream signaling pathways has emerged as an important factor in tumor growth and metastasis, as well as response to chemotherapy, targeted therapies and immunotherapy^19^. Therefore, it is of great significance to study ERSRGs in LUAD.

Herein, we identified a new prognostic model for LUAD patients based on the 7-ERSRGs signature and explored the potential therapeutic small molecule compounds. The endoplasmic reticulum stress-related model comprises seven ERSRGs, including PDX1, IGFBP1, DDIT4, PPP1R3G, CFTR, DERL3 and NUPR1. Researchers have found that model genes are closely associated with both carcinogenesis and anti-tumor therapy^10^. Pancreatic and duodenal homeobox 1 (PDX1) shifts from a tumor-suppressive to an oncogenic function after tumor transformation^20^. Interestingly, subsets of malignant cells lose PDX1 expression during the epithelial-to-mesenchymal transition (EMT), and PDX1 loss is associated with poor prognoses^20^. In our study, PDX1 tends to play a carcinogenic role, which may be due to the transformation of lung adenocarcinoma. A study found that insulin-like growth factor binding protein 1 (IGFBP1) accelerated hematogenous metastasis and led to poor survival in gastric cancer^21^. In vitro studies have suggested that DNA damage-inducible transcript 4 (DDIT4) could be a tumor suppressor or an oncogene in cancer, depending on the context^22,23^. The presence of protein phosphatase 1 regulatory subunit 3G (PPP1R3G) correlates with poor prognosis and immune infiltration in LUAD^24^. In murine and human intestinal cancers, the Cystic Fibrosis Transmembrane conductance Regulator (CFTR) acts as a tumor suppressor gene^25^. In gastric cancer, Derlin 3 (DERL3) acts as a tumor suppressor^26^. Nuclear protein‑1 (NUPR1) plays a role in promoting the proliferation of cancer cells by influencing cell cycle progression^27^. Generally speaking, since these 7 ERSRGs are critical to the development of tumors and are also involved in the development of treatment resistance, they have great potential as therapeutic targets or biomarkers in LUAD clinics.

According to the correlation results between seven-ERSRG expression and risk score, we found that Monocytes were the only immune cells that correlated with the expression of seven-ERSRG. Monocytes can bridge innate and adaptive immune responses and can affect the tumor microenvironment through various mechanisms that induce immune tolerance, angiogenesis, and increased dissemination of tumor cells. Yet monocytes can also give rise to antitumor effectors and activate antigen-presenting cells^28^. Previous studies showed that Monocytes play a key role in many pathological conditions and the outcome of diseases can be improved by controlling the numbers and functions of Monocytes^29^. The above results were consistent with our results, which suggested that Monocytes might provide a novel insight for treating patients with LUAD.

Based on the constructed model, we screened four potential drugs for the treatment of LUAD, including Phenformin, OSU-03012, GSK-650394, and KIN001-135. Researchers have found that phenformin inhibits cell proliferation and tumor growth more strongly than metformin in vitro and in vivo in various tumor types (e.g., breast, lung, glioblastoma, colon, melanoma, and prostate cancer)^30^. OSU-03012 is a novel celecoxib derivative that induces apoptosis in a variety of cancer cells without inhibiting cyclooxygenase-2^31^. A previous study found that GSK-650394 inhibited Influenza virus replication in cell models^32^. Moreover, we evaluated risk scores and the effectiveness of anticancer medications in our study, providing a fresh perspective on treating tumors and dealing with drug resistance^33,34^.

In conclusion, a new prognostic model is presented for ERSRGs in LUAD based on our study. This model was an independent predictor of OS in the train and test set, providing insight into how to predict prognosis for LUAD patients. However, there are some limitations in this study. On the one hand, we just derived the research data from the TCGA and GEO public databases. On the other hand, the function of the 7 ERSRGs has not been verified with biological experiments. A larger number of prospective real-world studies is needed to confirm its clinical efficacy. It is of great interest to target the 7-ERSRGs or small molecule compounds that are outlined above as possible components of combination therapies.

## Conclusion

A novel ERSRG signature for LUAD prognostic prediction was identified in the present study. Targeting ERSRGs might be a possible therapeutic approach for LUAD based on the small molecule compounds discovered in our study.

## Supplementary Information

Supplementary Information 1.

Supplementary Information 2.

Supplementary Information 3.

Supplementary Information 4.

Supplementary Information 5.

Supplementary Information 6.

Supplementary Information 7.

Supplementary Information 8.

Supplementary Information 9.

Supplementary Information 10.

Supplementary Information 11.

Supplementary Information 12.

Supplementary Information 13.

Supplementary Information 14.

Supplementary Information 15.

Supplementary Information 16.

## Data Availability

All data and R script in this study are available from the corresponding author upon reasonable request. Publicly available datasets were analyzed in this study, these can be found in The Cancer Genome Atlas (https://portal.gdc.cancer.gov/) and Molecular Signatures Database (http://www.gsea-msigdb.org/gsea/msigdb/index.jsp).
