# Supplementary figures and images for: Construction and validation of a prognostic model for lung adenocarcinoma based on endoplasmic reticulum stress-related genes
Source: Sci Rep. 2022 Nov 18;12:19857. doi: 10.1038/s41598-022-23852-z (PMC9674626; doi:10.1038/s41598-022-23852-z)

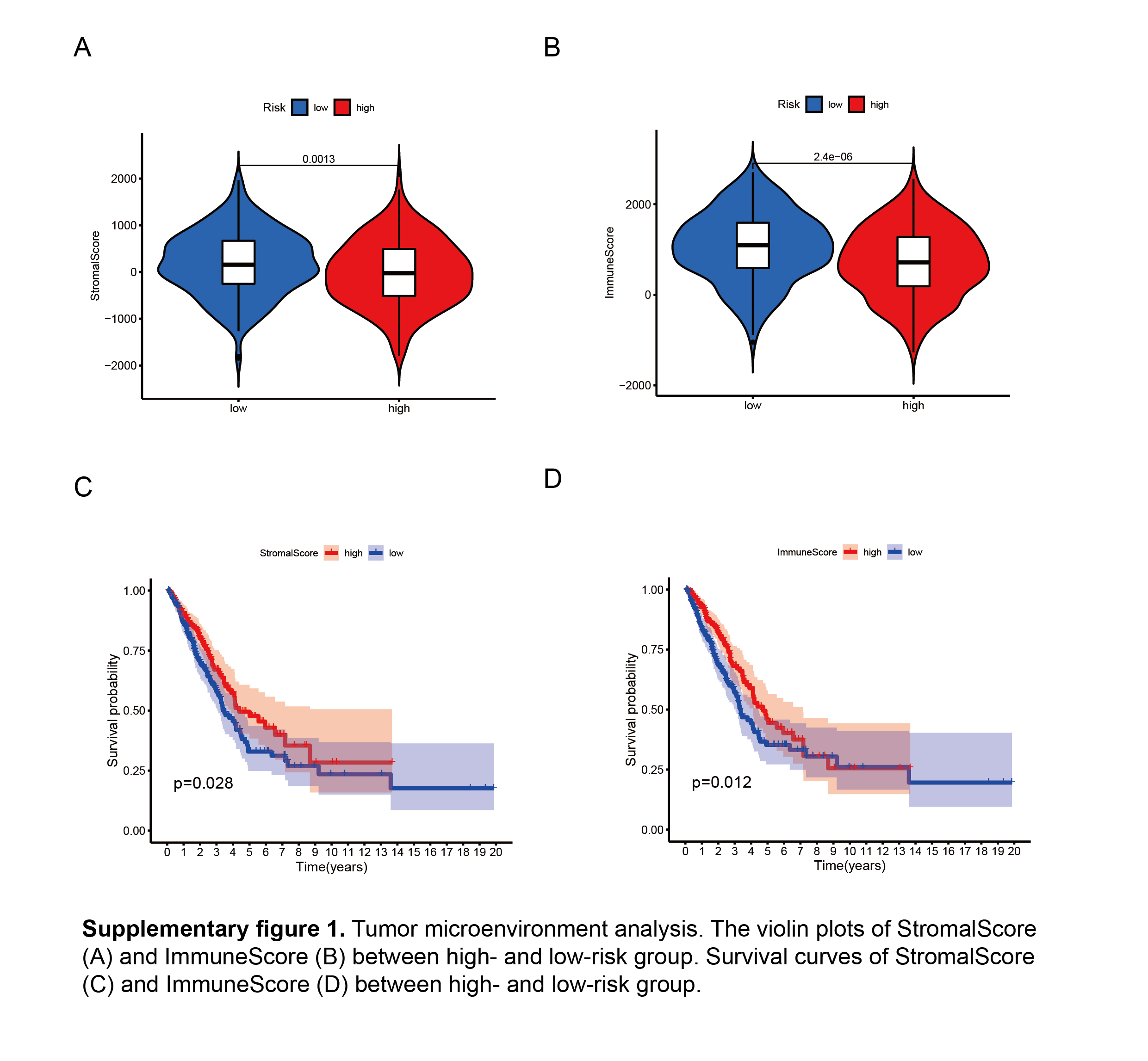

Supplement: Supplementary file 1 — Supplementary Information 1. [file 41598_2022_23852_MOESM1_ESM.jpg]

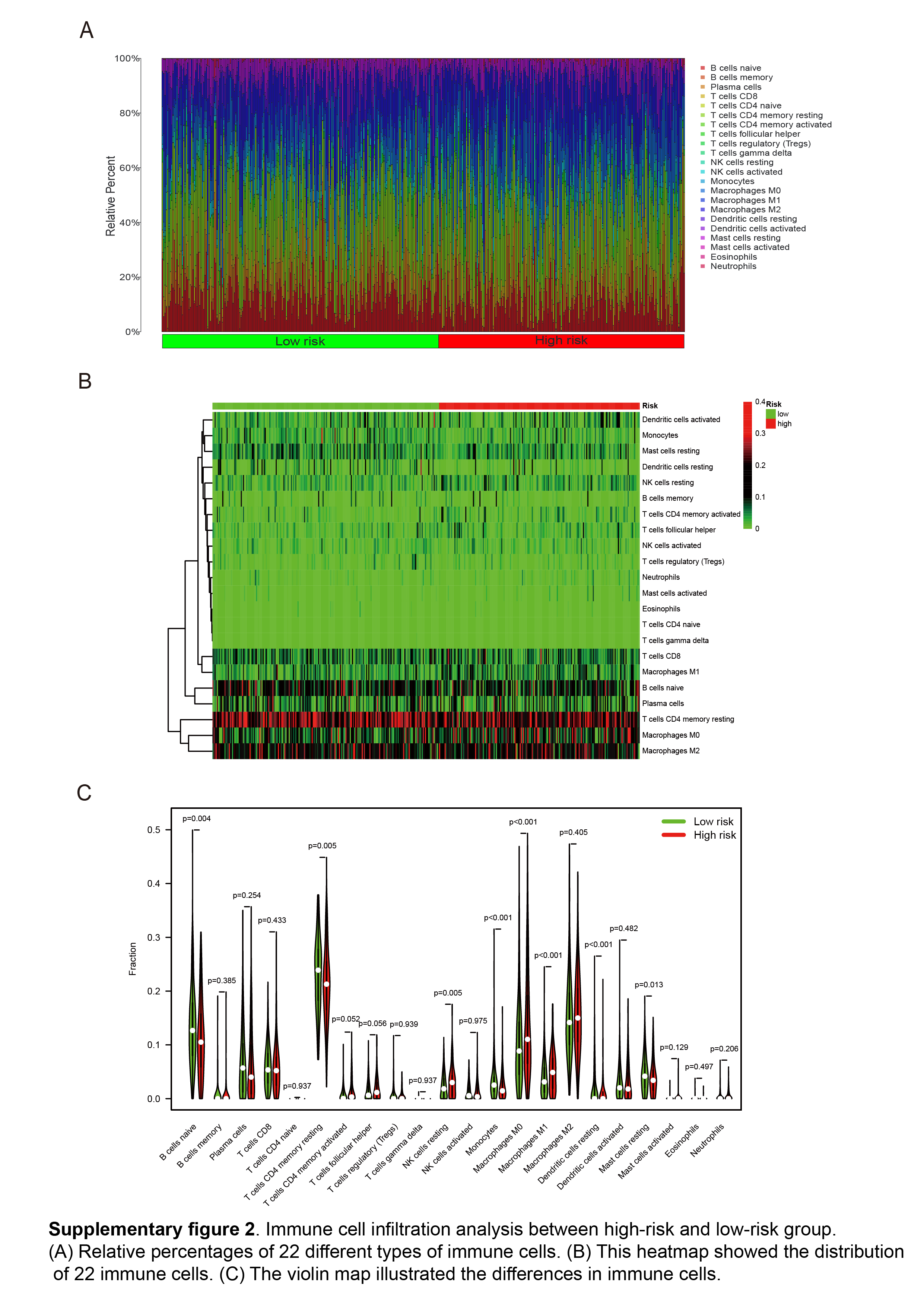

Supplement: Supplementary file 2 — Supplementary Information 2. [file 41598_2022_23852_MOESM2_ESM.jpg]

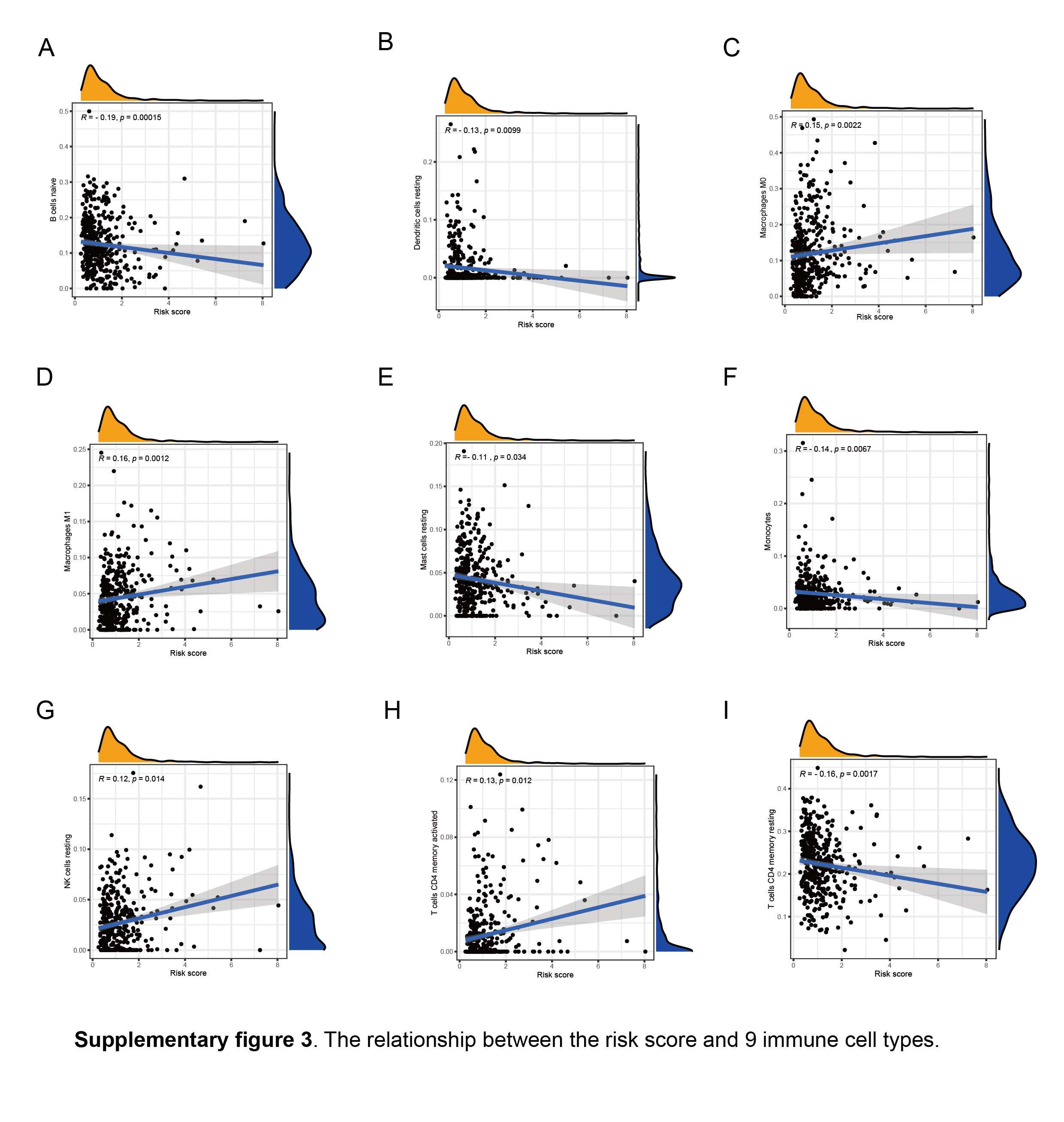

Supplement: Supplementary file 3 — Supplementary Information 3. [file 41598_2022_23852_MOESM3_ESM.jpg]

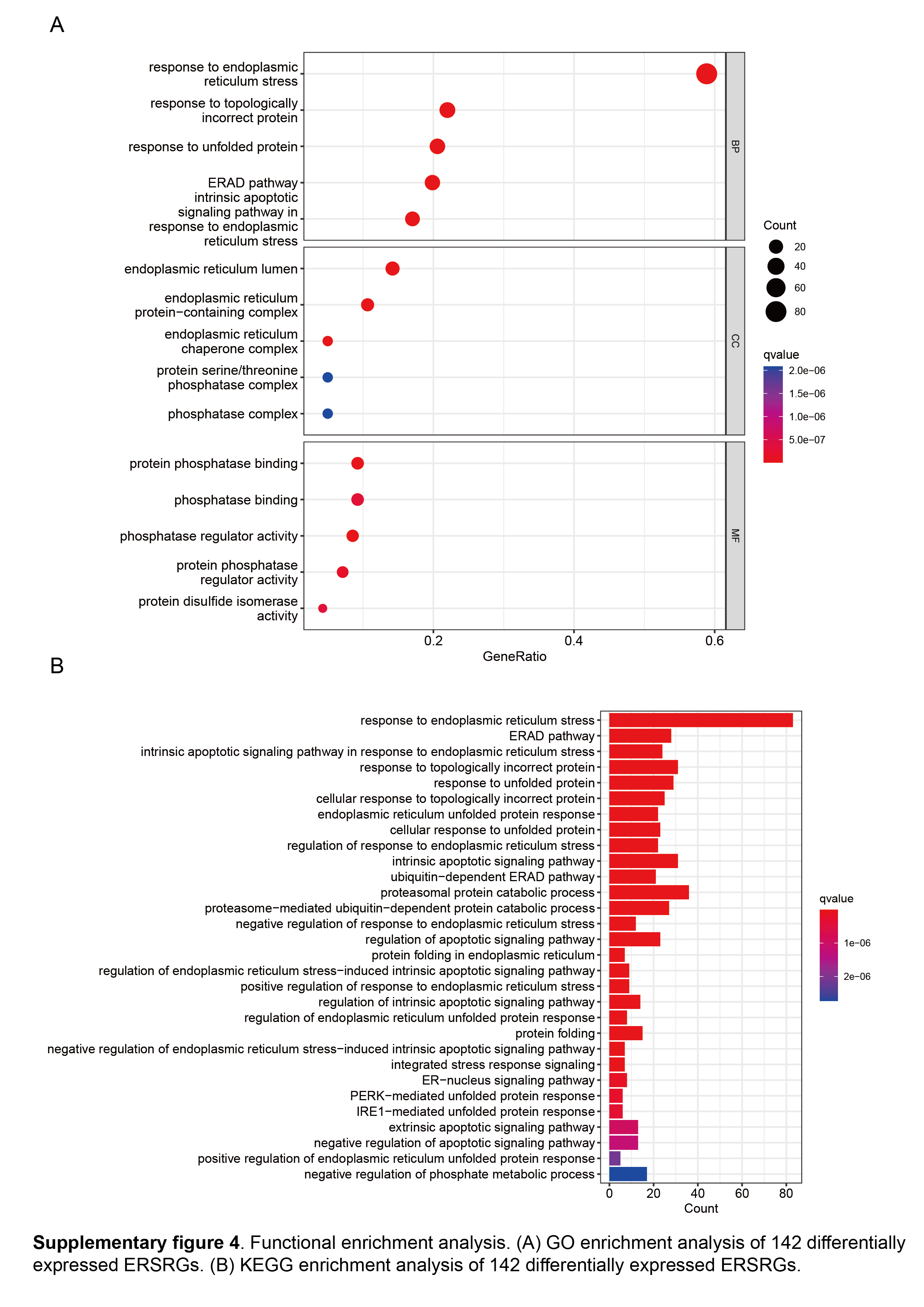

Supplement: Supplementary file 4 — Supplementary Information 4. [file 41598_2022_23852_MOESM4_ESM.jpg]

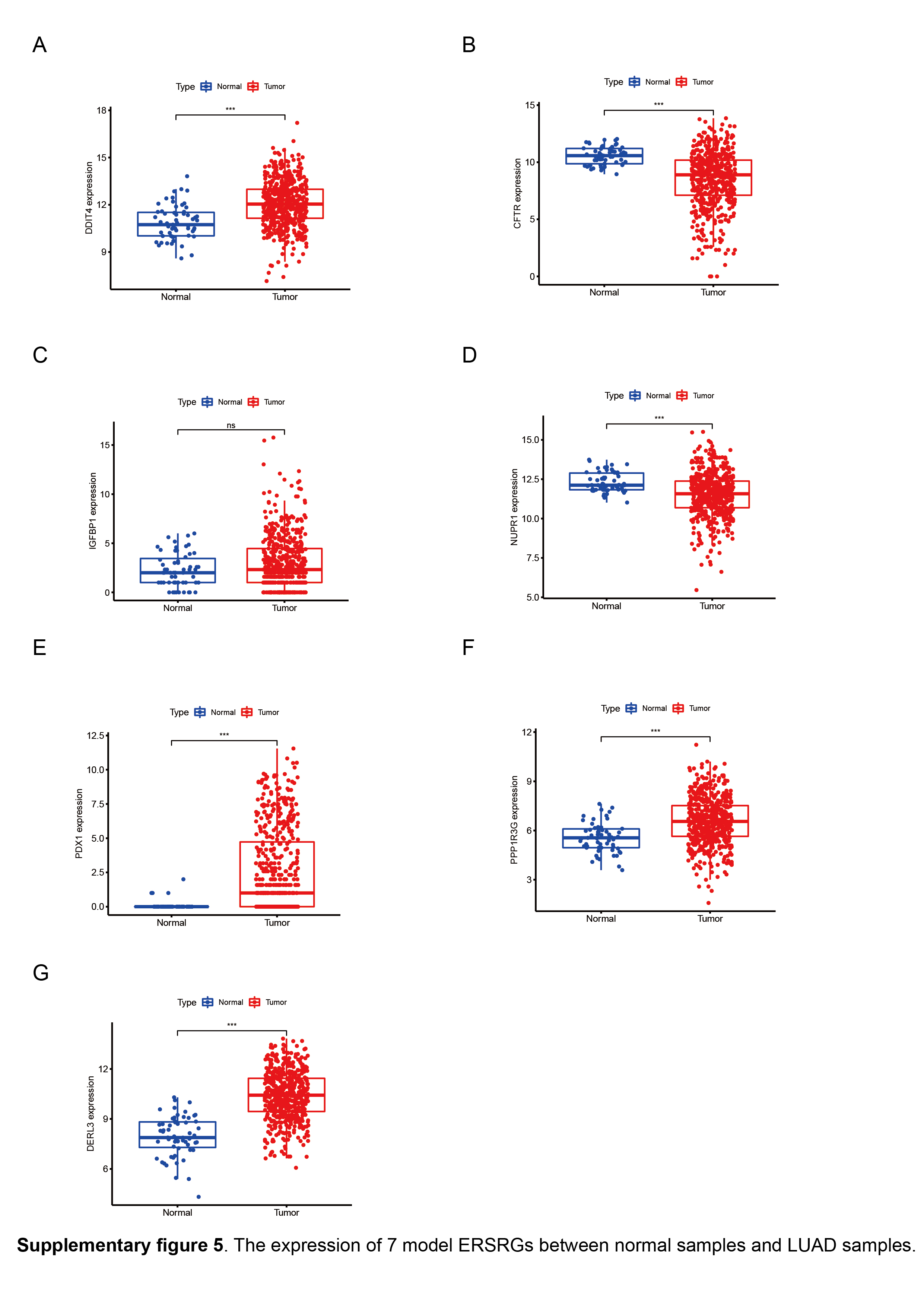

Supplement: Supplementary file 5 — Supplementary Information 5. [file 41598_2022_23852_MOESM5_ESM.jpg]

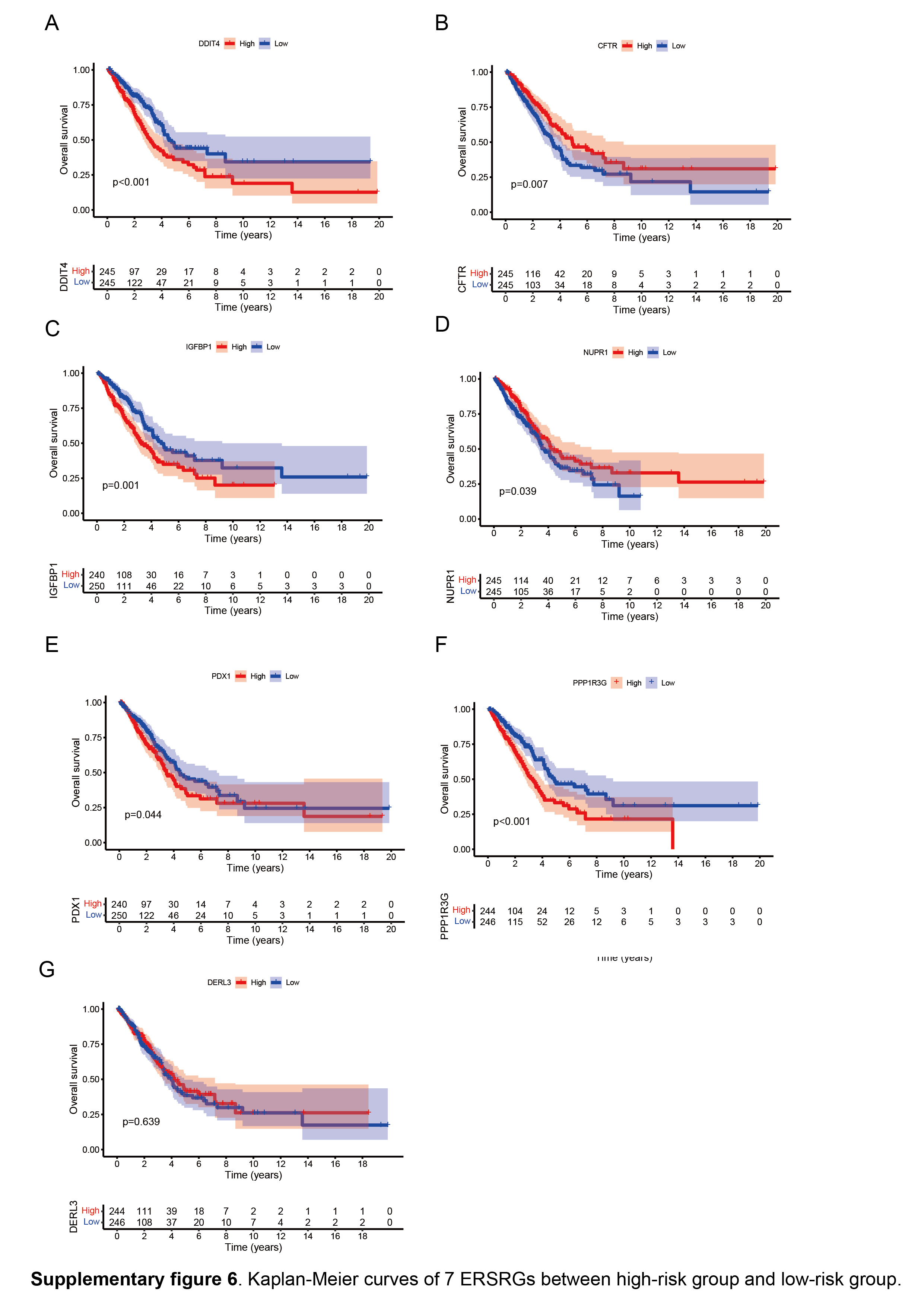

Supplement: Supplementary file 6 — Supplementary Information 6. [file 41598_2022_23852_MOESM6_ESM.jpg]

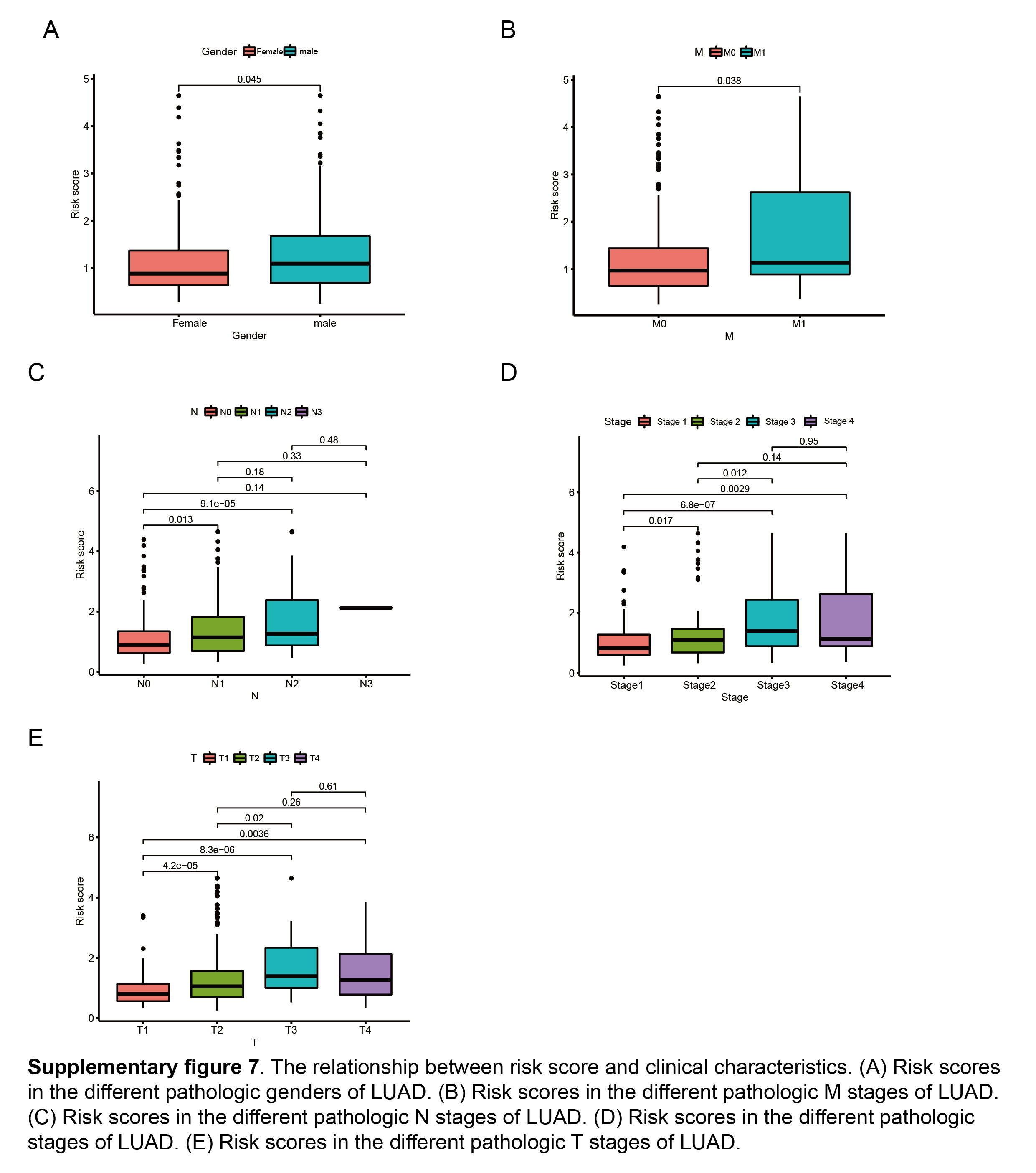

Supplement: Supplementary file 7 — Supplementary Information 7. [file 41598_2022_23852_MOESM7_ESM.jpg]

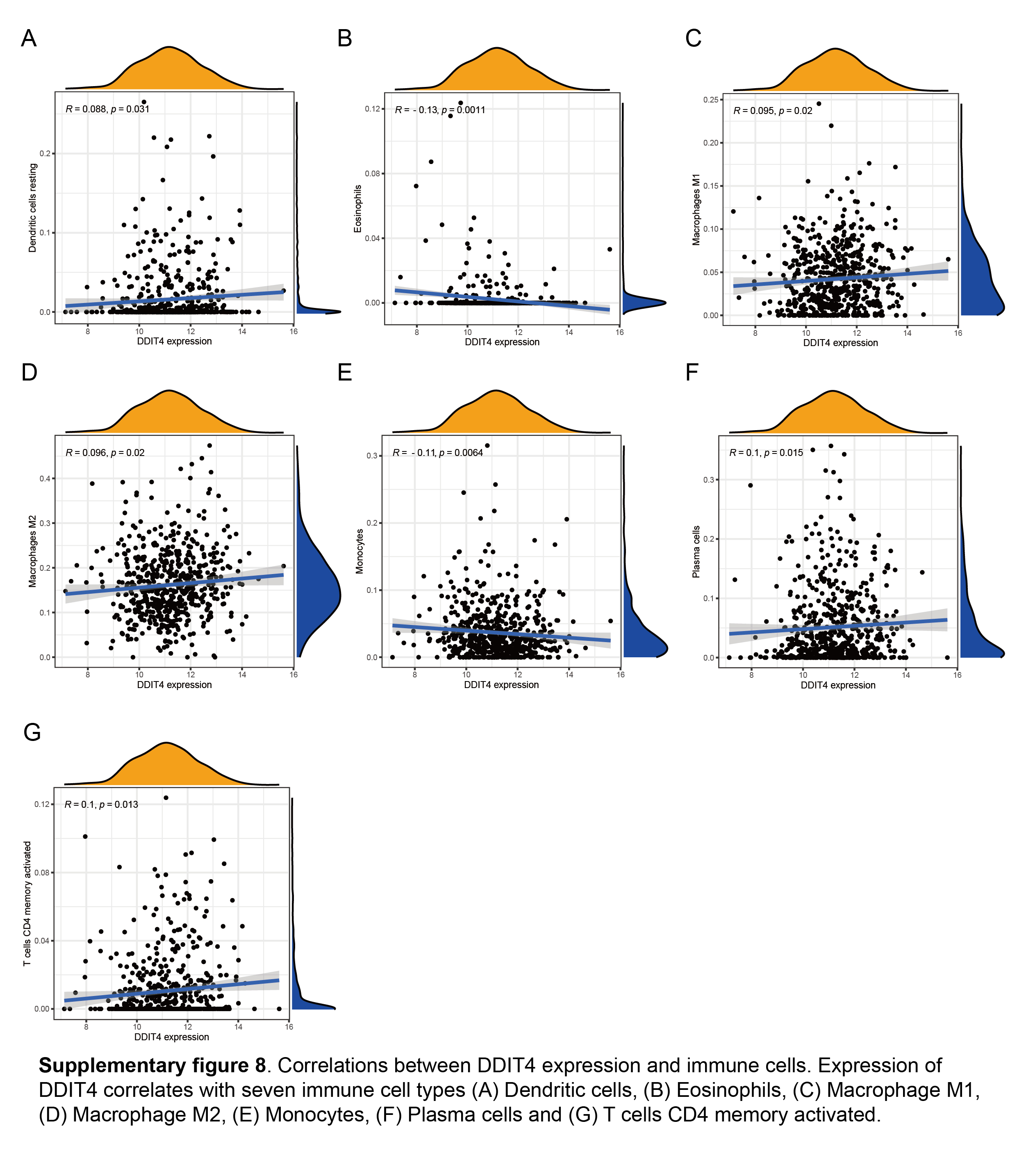

Supplement: Supplementary file 8 — Supplementary Information 8. [file 41598_2022_23852_MOESM8_ESM.jpg]

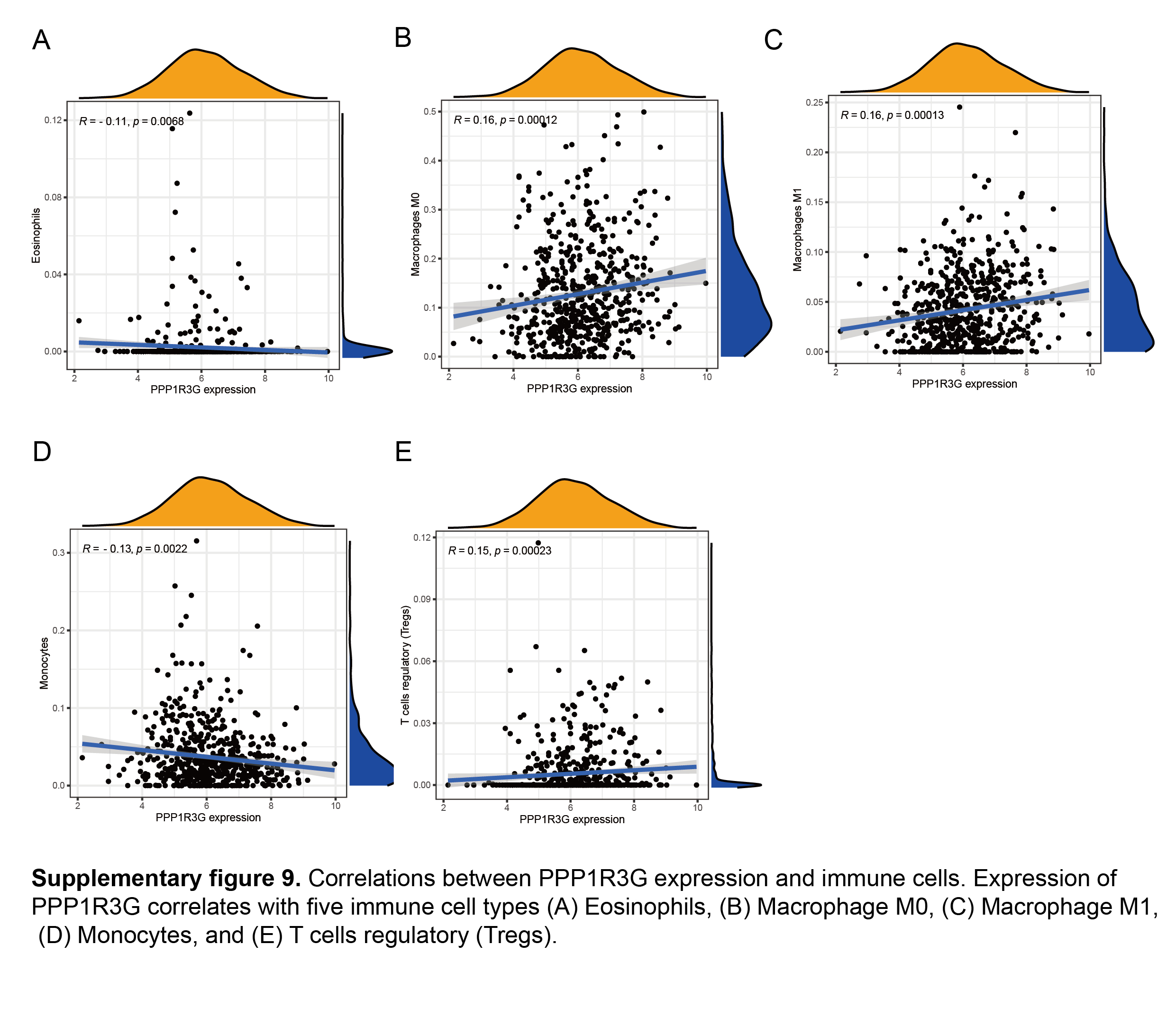

Supplement: Supplementary file 9 — Supplementary Information 9. [file 41598_2022_23852_MOESM9_ESM.jpg]

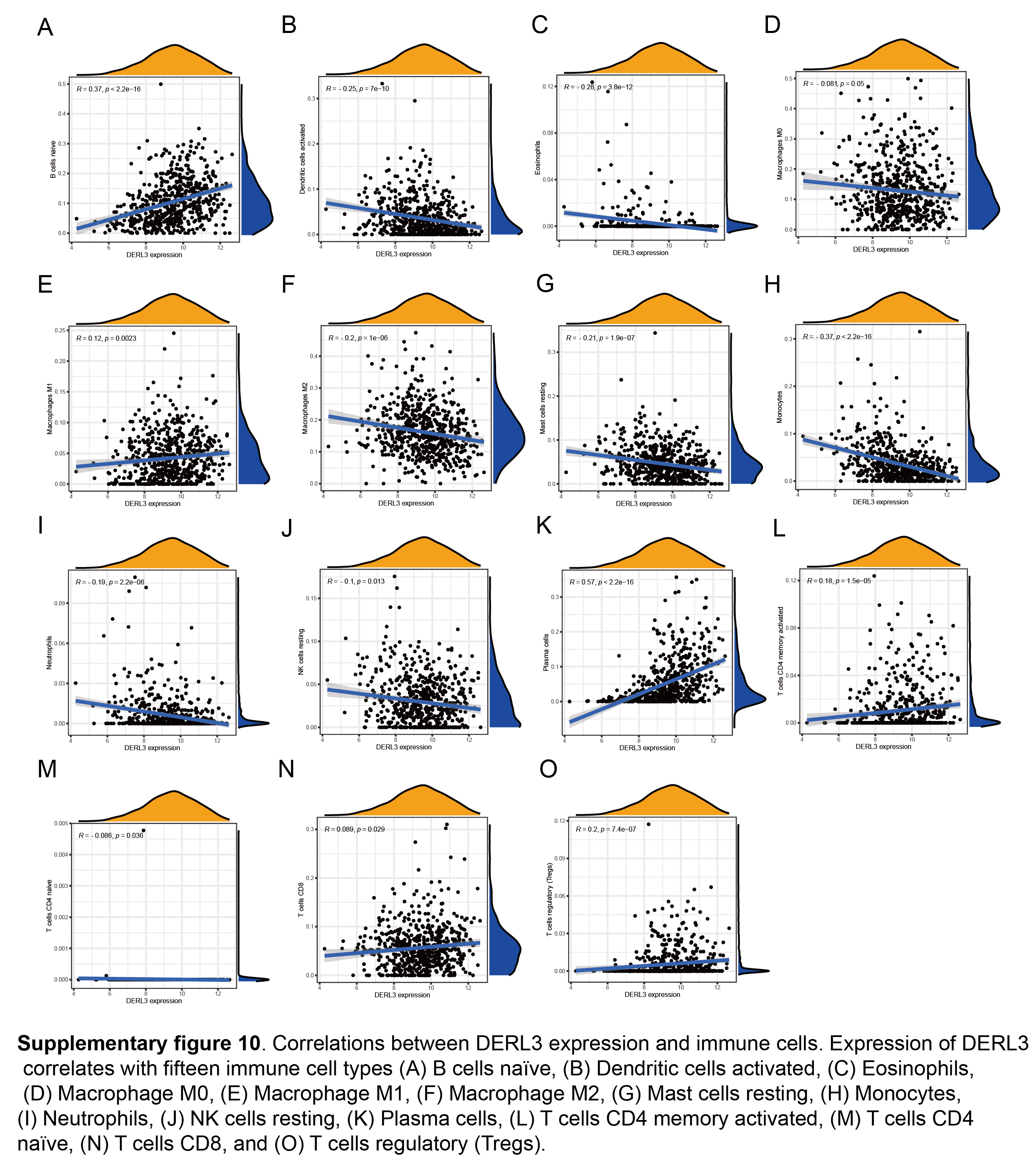

Supplement: Supplementary file 10 — Supplementary Information 10. [file 41598_2022_23852_MOESM10_ESM.jpg]

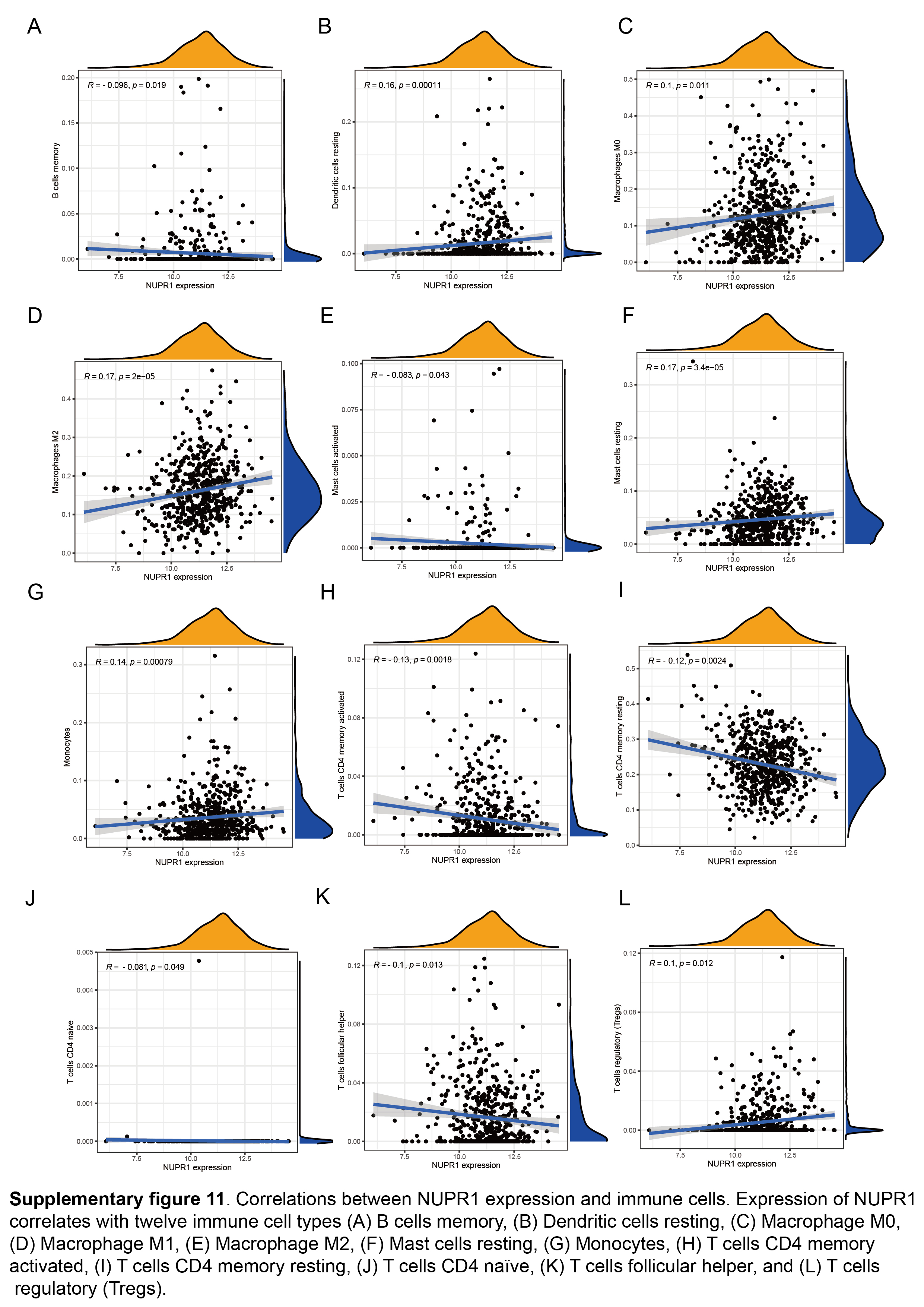

Supplement: Supplementary file 11 — Supplementary Information 11. [file 41598_2022_23852_MOESM11_ESM.jpg]

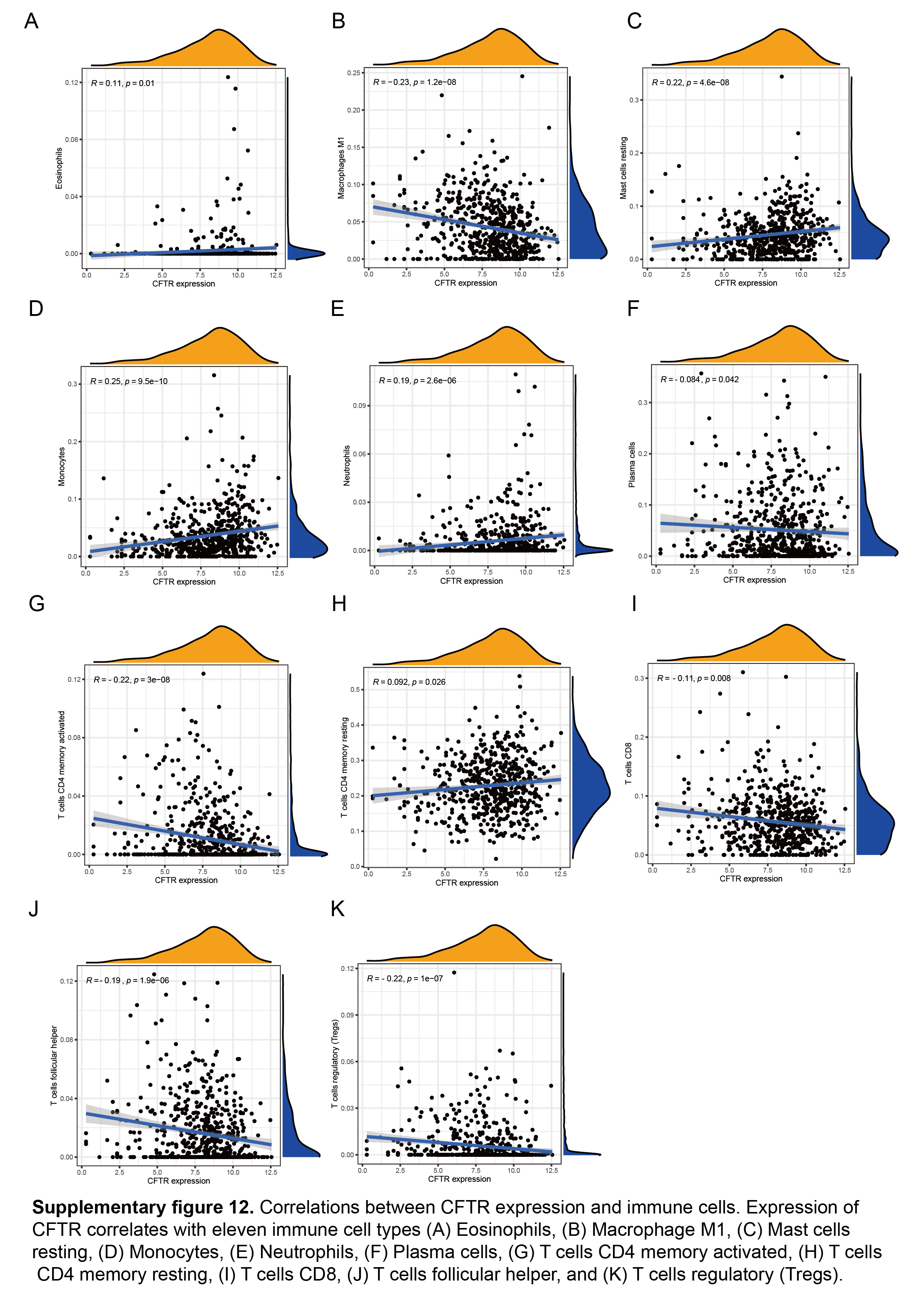

Supplement: Supplementary file 12 — Supplementary Information 12. [file 41598_2022_23852_MOESM12_ESM.jpg]

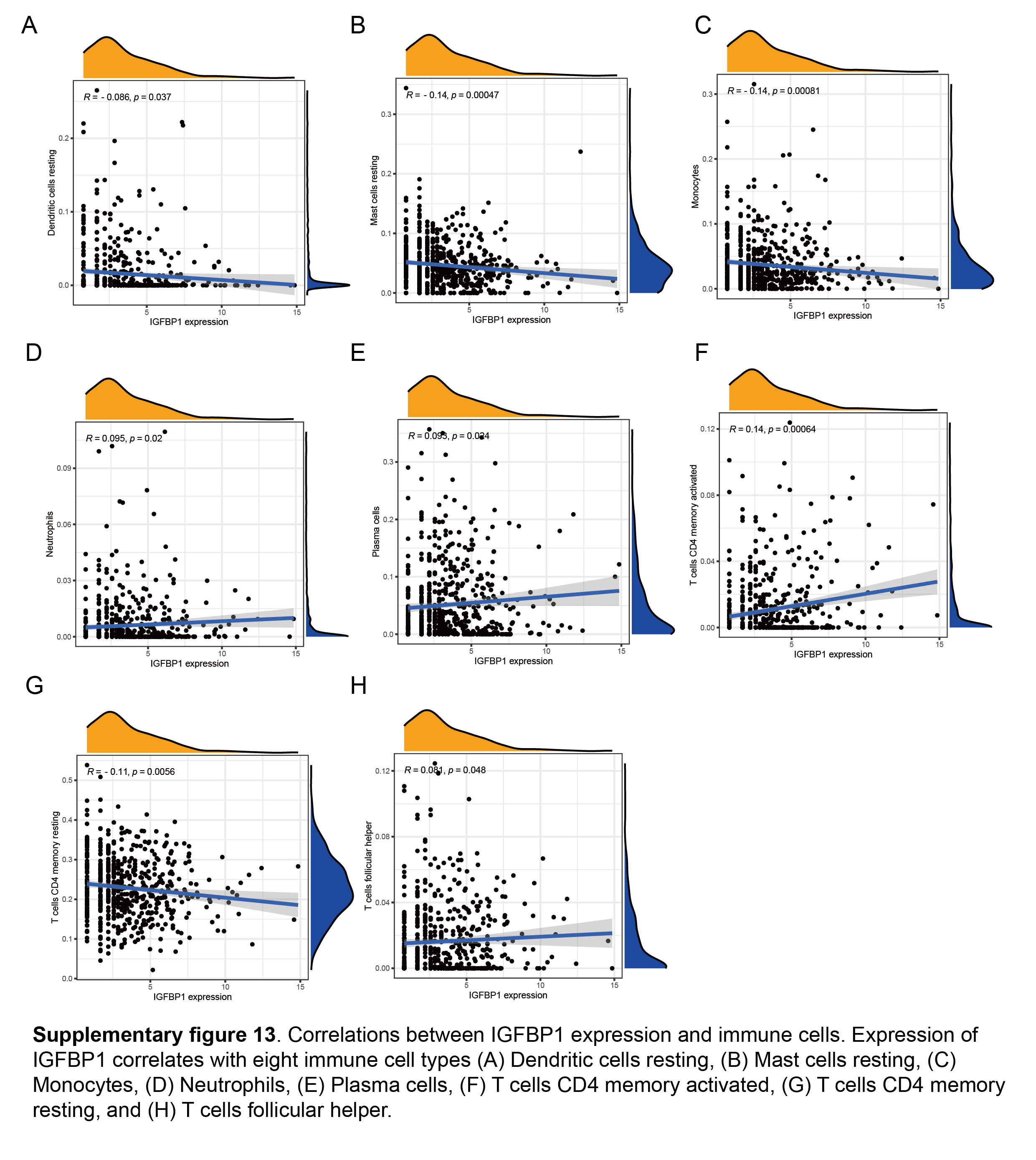

Supplement: Supplementary file 13 — Supplementary Information 13. [file 41598_2022_23852_MOESM13_ESM.jpg]

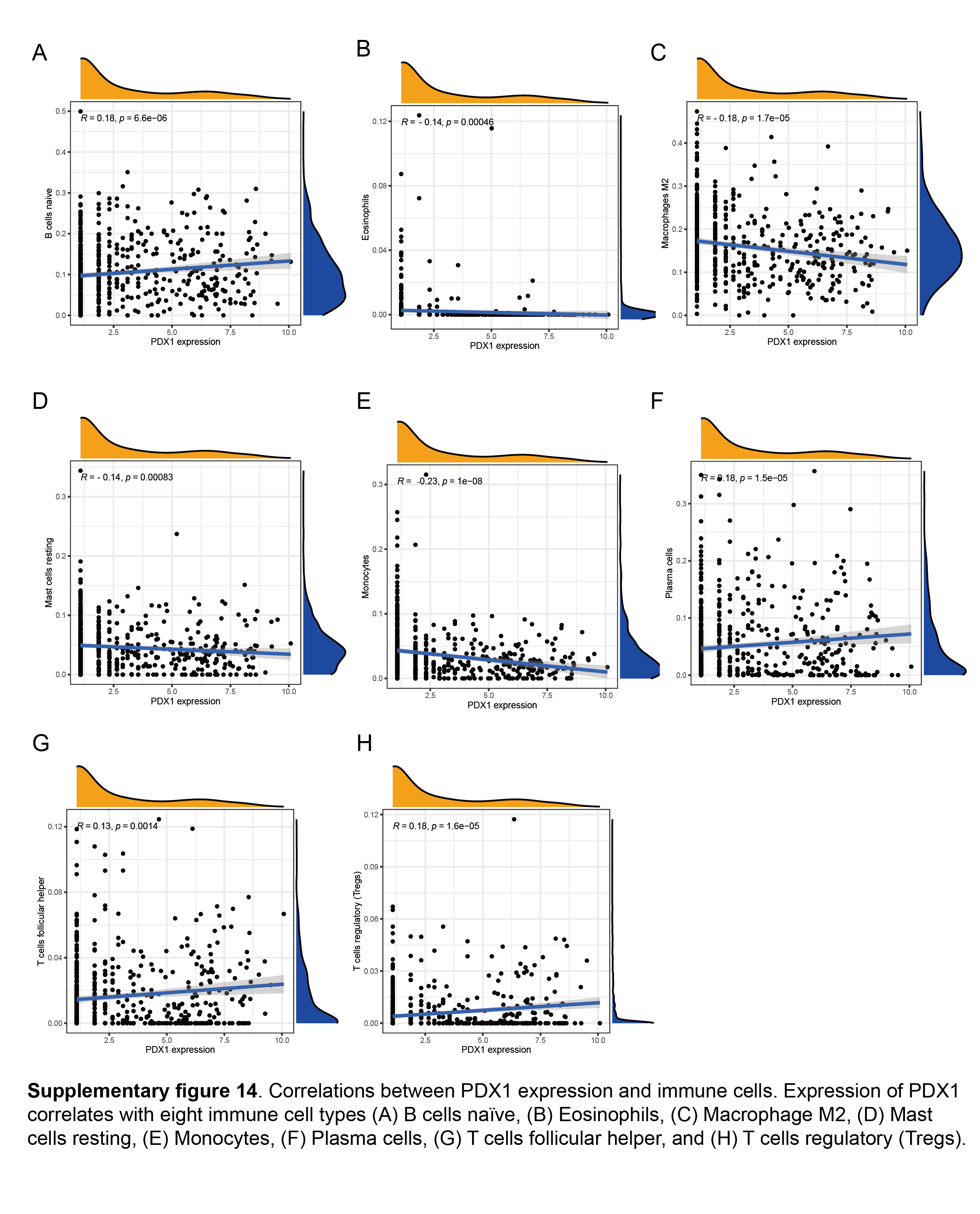

Supplement: Supplementary file 14 — Supplementary Information 14. [file 41598_2022_23852_MOESM14_ESM.jpg]
