# Supplementary material for: Construction and validation of a prognostic model for lung adenocarcinoma based on endoplasmic reticulum stress-related genes
Source: Sci Rep. 2022 Nov 18;12:19857. doi: 10.1038/s41598-022-23852-z (PMC9674626; doi:10.1038/s41598-022-23852-z)
Supplement: Supplementary file 16 — Supplementary Information 16. [file 41598_2022_23852_MOESM16_ESM.docx]

**Supplementary figure 1. Tumor microenvironment analysis.** The violin plots of StromalScore (A) and ImmuneScore (B) between high- and low-risk group. Survival curves of StromalScore (C) and ImmuneScore (D) between high- and low-risk group.

**Supplementary figure 2**. **Immune cell infiltration analysis between high-risk and low-risk group.** (A) Relative percentages of 22 different types of immune cells. (B) This heatmap shows the distribution of 22 immune cells. (C) The violin map illustrated the differences in immune cells.

**Supplementary figure 3**. **The relationship between the risk score and 9 immune cell types.**

**Supplementary figure 4. Functional enrichment analysis.** (A) GO enrichment analysis of 142 differentially expressed ERSRGs. (B) KEGG enrichment analysis of 142 differentially expressed ERSRGs.

**Supplementary figure 5.** **The expression of 7 model ERSRGs between normal samples and LUAD samples.**

**Supplementary figure 6.** **Kaplan-Meier curves of 7 model ERSRGs between high-risk group and low-risk group.**

**Supplementary figure 7.** **The relationship between risk score and clinical characteristics.** (A) Risk scores in the different pathologic genders of LUAD. (B) Risk scores in the different pathologic M stages of LUAD. (C) Risk scores in the different pathologic N stages of LUAD. (D) Risk scores in the different pathologic stages of LUAD. (E) Risk scores in the different pathologic T stages of LUAD.

**Supplementary figure 8.** **Correlations between DDIT4 expression and immune cells.** Expression of DDIT4 correlates with seven immune cell types. (A) Dendritic cells, (B) Eosinophils, (C) Macrophage M1, (D) Macrophage M2, (E) Monocytes, (F) Plasma cells and (G) T cells CD4 memory activated.

**Supplementary figure 9.** **Correlations between PPP1R3G expression and immune cells.** Expression of PPP1R3G correlates with five immune cell types. (A) Eosinophils, (B) Macrophage M0, (C) Macrophage M1, (D) Monocytes, and (E) T cells regulatory (Tregs).

**Supplementary figure 10.** **Correlations between DERL3 expression and immune cells.** Expression of DERL3 correlates with fifteen immune cell types (A) B cells naïve, (B) Dendritic cells activated, (C) Eosinophils, (D) Macrophage M0, (E) Macrophage M1, (F) Macrophage M2, (G) Mast cells resting, (H) Monocytes, (I) Neutrophils, (J) NK cells resting, (K) Plasma cells, (L) T cells CD4 memory activated, (M) T cells CD4 naïve, (N) T cells CD8, and (O) T cells regulatory (Tregs).

**Supplementary figure 11.** **Correlations between NUPR1 expression and immune cells.** Expression of NUPR1 correlates with twelve immune cell types (A) B cells memory, (B) Dendritic cells resting, (C) Macrophage M0, (D) Macrophage M1, (E) Macrophage M2, (F) Mast cells resting, (G) Monocytes, (H) T cells CD4 memory activated, (I) T cells CD4 memory resting, (J) T cells CD4 naïve, (K) T cells follicular helper, and (L) T cells regulatory (Tregs).

**Supplementary figure 12.** **Correlations between CFTR expression and immune cells.** Expression of CFTR correlates with eleven immune cell types (A) Eosinophils, (B) Macrophage M1, (C) Mast cells resting, (D) Monocytes, (E) Neutrophils, (F) Plasma cells, (G) T cells CD4 memory activated, (H) T cells CD4 memory resting, (I) T cells CD8, (J) T cells follicular helper, and (K) T cells regulatory (Tregs).

**Supplementary figure 13.** **Correlations between IGFBP1 expression and immune cells.** Expression of IGFBP1 correlates with eight immune cell types (A) Dendritic cells resting, (B) Mast cells resting, (C) Monocytes, (D) Neutrophils, (E) Plasma cells, (F) T cells CD4 memory activated, (G) T cells CD4 memory resting, and (H) T cells follicular helper.

**Supplementary figure 14. Correlations between PDX1 expression and immune cells.** Expression of PDX1 correlates with eight immune cell types (A) B cells naïve, (B) Eosinophils, (C) Macrophage M2, (D) Mast cells resting, (E) Monocytes, (F) Plasma cells, (G) T cells follicular helper, and (H) T cells regulatory (Tregs).

**Supplementary Table 1.** List of differentially expressed ERSRGs.
